# Factor-inhibiting HIF (FIH) promotes lung cancer progression

**DOI:** 10.1172/jci.insight.167394

**Published:** 2023-10-23

**Authors:** Ana García-del Río, Endika Prieto-Fernández, Leire Egia-Mendikute, Asier Antoñana-Vildosola, Borja Jimenez-Lasheras, So Young Lee, Adrián Barreira-Manrique, Samanta Romina Zanetti, Ander de Blas, Paloma Velasco-Beltrán, Alexandre Bosch, Ana M. Aransay, Asis Palazon

**Affiliations:** 1 Cancer Immunology and Immunotherapy Lab, Center for Cooperative Research in Biosciences (CIC bioGUNE), Basque Research and Technology Alliance (BRTA), Derio, Bizkaia, Spain.; 2 Centro de Investigación Biomédica en Red de Enfermedades Hepáticas y Digestivas (CIBEREHD), Instituto de Salud Carlos III, Madrid, Spain.; 3 Genome Analysis Platform, CIC bioGUNE, Bizkaia Technology Park, Derio, Bizkaia, Spain.; 4 Ikerbasque, Basque Foundation for Science, Bilbao, Spain.

**Keywords:** Cell Biology, Cancer, Cellular immune response, Hypoxia

## Abstract

Factor-inhibiting HIF (FIH) is an asparagine hydroxylase that acts on hypoxia-inducible factors (HIFs) to control cellular adaptation to hypoxia. FIH is expressed in several tumor types, but its impact in tumor progression remains largely unexplored. We observed that FIH was expressed on human lung cancer tissue. Deletion of *FIH* in mouse and human lung cancer cells resulted in an increased glycolytic metabolism, consistent with increased HIF activity. *FIH*-deficient lung cancer cells exhibited decreased proliferation. Analysis of RNA-Seq data confirmed changes in the cell cycle and survival and revealed molecular pathways that were dysregulated in the absence of *FIH*, including the upregulation of angiomotin (*Amot*), a key component of the Hippo tumor suppressor pathway. We show that *FIH*-deficient tumors were characterized by higher immune infiltration of NK and T cells compared with FIH competent tumor cells. In vivo studies demonstrate that *FIH* deletion resulted in reduced tumor growth and metastatic capacity. Moreover, high FIH expression correlated with poor overall survival in non–small cell lung cancer (NSCLC). Our data unravel FIH as a therapeutic target for the treatment of lung cancer.

## Graphical abstract

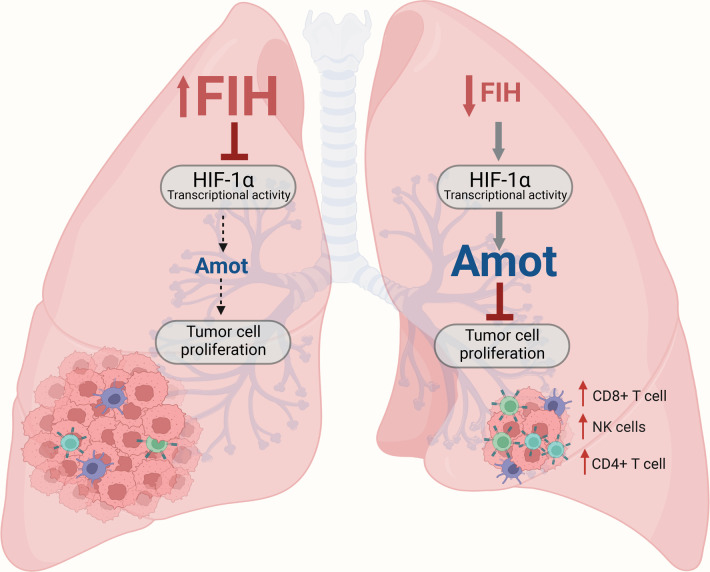

## Introduction

Lung cancer is the leading cause of global cancer-related death, with a low overall 5-year survival rate (1). Non–small cell lung cancer (NSCLC) is the most prevalent histological subtype of lung cancer, and there is an urgent medical need to both understand the biology of the disease and develop novel efficacious therapies (2, 3).

Immunotherapy, either alone or in combination with other therapies, promotes T cell function to improve clinical outcome. Checkpoint blockade with monoclonal antibodies attains significant responses in lung cancer, but only a fraction of patients is eligible or respond to immunotherapy (4, 5). The tumor microenvironment (TME) in solid tumors is known to limit T cell functionality through a variety of mechanisms (6, 7). Hypoxia is a common feature present in the TME of solid tumors, playing a key role in tumor progression and immunity (8). Cellular adaptation to hypoxia is regulated by hypoxia-inducible factors (HIFs), which are transcription factors that control pathways related to metabolism, proliferation, inflammation, survival, and angiogenesis, among others (9–13). For these reasons, several drug candidates targeting this pathway are being developed for the treatment of cancer (14).

The HIF molecular pathway is regulated by prolyl hydroxylases (PHDs) and the asparaginyl hydroxylase factor-inhibiting HIF (FIH). HIF accumulation and activity are regulated by posttranslational modifications that depend on the presence of O_2_. PHDs catalyze prolyl hydroxylation of HIF, triggering degradation by the action of the von Hippel-Lindau (VHL) E3 ubiquitin ligase complex and subsequent HIF ubiquitination and proteasomal degradation (15–17). The other main layer of regulation of HIF is mediated by FIH hydroxylation on a key asparaginyl residue (Asn803) (18). This enzymatic reaction suppresses HIF transcriptional activity by preventing its binding to the transcriptional coactivators p300/CBP (19–22). This reaction requires iron (Fe II), 2-oxoglutarate (2-OG), and molecular O_2_ (23, 24). In the presence of O_2_, PHDs and FIH are active, resulting in repression of the hypoxia pathway. When O_2_ levels drop, PHDs and FIH become catalytically inactive, resulting in the lack of hydroxylation of HIF, driving HIF accumulation, nuclear translocation, and downstream transcriptional activation (25). FIH requires more severe hypoxic conditions than the PHDs to become inactive (26–28).

FIH can hydroxylate asparagine residues on other proteins outside the HIF pathway (29), including members of the ankyrin repeat domain (ARD) protein family (30–33), such as NF-κB inhibitor α (Iκbα) (30, 34), Notch (31, 35), ASPP2 (32, 36), TRPV3 (37), ASB4 (38), and HACE1 (38, 39). Moreover, non–ARD-containing proteins have also been reported to be hydroxylated by FIH: Cezanne (OTUD7B) (40) and OTUB1 (41, 42).

FIH protein is found in both healthy and malignant tissues (43–45). In addition, FIH subcellular localization is mainly cytoplasmic (43) and, in some cases, can translocate to the nucleus associated with HIF (46). The most studied biological function of FIH is the regulation of metabolism in the context of adaptation to hypoxia (45, 47–49).

The impact of FIH in cancer biology remains largely unexplored. However, some studies have shown that FIH can influence tumor growth in a variety of cancers: renal cell carcinoma (50, 51), colon cancer (52–55), melanoma (53), breast cancer (39, 56), osteosarcoma (57), pancreatic endocrine tumors (58), glioblastoma multiforme (59), hepatocellular carcinoma (60), and NSCLC (61, 62). FIH has also been shown to influence vascular endothelial cell survival (63) and the metastatic process (64).

In this study, we investigated the role of FIH in lung cancer, demonstrating its protumorigenic nature and revealing it as a potential therapeutic target.

## Results

### FIH is expressed in lung cancer.

**Figure 1:**
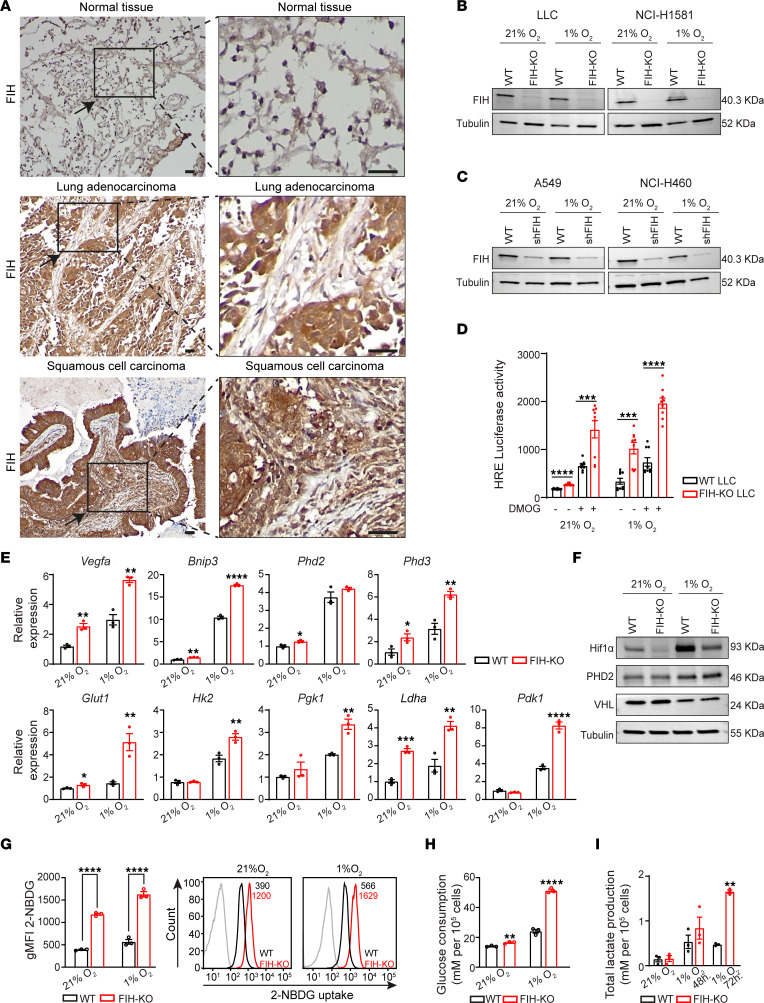
FIH is expressed in lung cancer tissue and regulates HIF-driven metabolism. (**A**) Representative IHC images showing FIH expression (peroxidase-DAB brown staining) in normal tissue and lung cancer. Enlarged images are shown in the insets. Scale bars: 500 μm. (**B**) Western blot showing FIH expression in the indicated WT or *FIH*-KO cell lines generated by CRISPR/Cas9. (**C**) Western blot showing FIH expression in the indicated WT or *FIH*-knockdown human cell lines generated by short hairpin RNA (shRNA). (**D**) HRE luciferase reporter activity assay performed with LLC cells cultured in the presence or absence of 1 mM DMOG under the indicated conditions (*n* = 3, technical replicates are shown, unpaired *t* test). (**E**) Relative expression of the indicated HIF target genes in WT or *FIH*-KO LLC cells (*n* = 3, unpaired *t* test). (**F**) Immunoblotting showing HIF1A, PHD2, VHL, and tubulin expression in WT or *FIH*-KO LLC cells. (**G**) Flow cytometry analysis of glucose uptake (2-NBDG) in WT (black) or *FIH*-KO (red) LLC cells cultured under normoxia or hypoxia for 48 hours. A representative histogram indicating the geometric mean fluorescent intensity (gMFI) value for each condition (unstained, gray; WT, black or *FIH*-KO, red) is shown (*n* = 3, 2-way ANOVA). (**H**) Cell glucose consumption in WT or *FIH*-KO LLC cells cultured under normoxia or hypoxia for 48 hours measured by a colorimetric enzymatic assay (*n* = 3, unpaired *t* test). (**I**) Lactate production in WT or *FIH*-KO LLC cells cultured under normoxia or hypoxia for 48 hours and 72 hours measured by a colorimetric enzymatic assay (*n* = 3, unpaired *t* test). Cells were cultured for 16 hours under normoxia or hypoxia in **B**–**F**. Data are shown as mean ± SEM. *P ≤ 0.05, **P ≤ 0.01, ***P ≤ 0.001, and ****P ≤ 0.0001.

We first assessed the expression of FIH (*HIF1AN* gene) across different tissues and tumor types at the transcriptional level. *FIH* is found in a variety of healthy organs (Supplemental Figure 1A; supplemental material available online with this article; https://doi.org/10.1172/jci.insight.167394DS1). Focusing on lung cancer, we interrogated the levels of *FIH* in lung solid tumors compared with healthy tissue, showing that *FIH* is present at the transcriptional level in both lung adenocarcinoma (LUAD) and lung squamous cell carcinoma (LUSC) (Supplemental Figure 1B). Additionally, *FIH* expression was confirmed in several lung cancer cell lines (Supplemental Figure 1C). With the objective of characterizing the intrinsic role of FIH in lung cancer cells, we selected a panel of human cell lines (A549, NCI-460, and NCI-H1581) and a murine cell line (Lewis lung carcinoma [LLC]). In both human and mouse cell lines, hypoxia induced the expression of HIF target genes (*VEGFA*, *PHD2*) but had no effect on the levels of expression of FIH at the transcriptional or protein levels, measured at different time points and O_2_ concentrations (Supplemental Figure 2, A–D). In order to confirm that FIH is present on lung tumor tissue at the protein level, we stained tissue microarrays from patients with lung cancer (*n* = 25), including LUAD (*n* = 9) and LUSC (*n* = 16), as well as normal tissue (*n* = 4). Figure 1A shows that FIH expression is abundant in both the cytoplasm and nucleus of lung cancer cells. We found that FIH is present in all lung tumor samples tested, with varying degrees of expression (Figure 1A and Supplemental Figure 3). In addition, FIH is mainly found in the cytoplasm of the lung cancer cells LLC, NCI-H1581, A549, and NCI-H460 (Supplemental Figure 4). These findings demonstrate that FIH is expressed in both normal and tumor lung tissues.

### FIH influences the metabolism of lung cancer cells.

Immunoblotting revealed that FIH protein is present in murine and human lung cancer cell lines (Figure 1, B and C). We then assessed the functional impact of *FIH* deletion on cellular metabolism and hypoxia adaptation. To that end, we first generated several *FIH*-KO or *FIH*-knockdown cell lines using CRISPR/Cas9 (Figure 1B) or short hairpin RNA (Figure 1C) methodologies, respectively. *FIH*-KO cells presented increased HIF activity, measured by a hypoxia-response element (HRE) luciferase reporter assay (Figure 1D and Supplemental 5A). *FIH* deletion increased the expression of HIF target genes that drive adaptation to hypoxia (*Vegfa*, *Bnip3*, *Phd2*, *Phd3)*, glucose metabolism (*Glut1*, *Hk2*, *Pgk1*, *Ldha*), and metabolic reprogramming (*Pdk1*) (Figure 1E). In addition, we showed that the observed increase in HIF transcriptional activity upon *FIH* deletion was accompanied by reduced levels of HIF-1α (HIF1A) under both normoxia and hypoxia (Figure 1F). Of note, hypoxia altered the expression of PHD2 and VHL, but *FIH* deletion had no affect on their total protein levels in either condition (Figure 1F). In order to study the metabolic consequences of *FIH* deletion, we assessed glucose consumption and lactate production in WT or *FIH*-KO cell lines after culture under normoxia or hypoxia for different time points. Loss of *FIH* increased cellular glucose uptake (Figure 1, G and H) and lactate production (Figure 1I), a phenotype that was more evident in hypoxia. Similar findings were observed with the human cell line NCI-H1581 (Supplemental Figure 5, A–D). Overall, these results demonstrate that FIH is a functional regulator of HIF activity in lung cancer cells.

### The absence of FIH in lung tumor cells impairs proliferation and promotes apoptosis.

**Figure 2:**
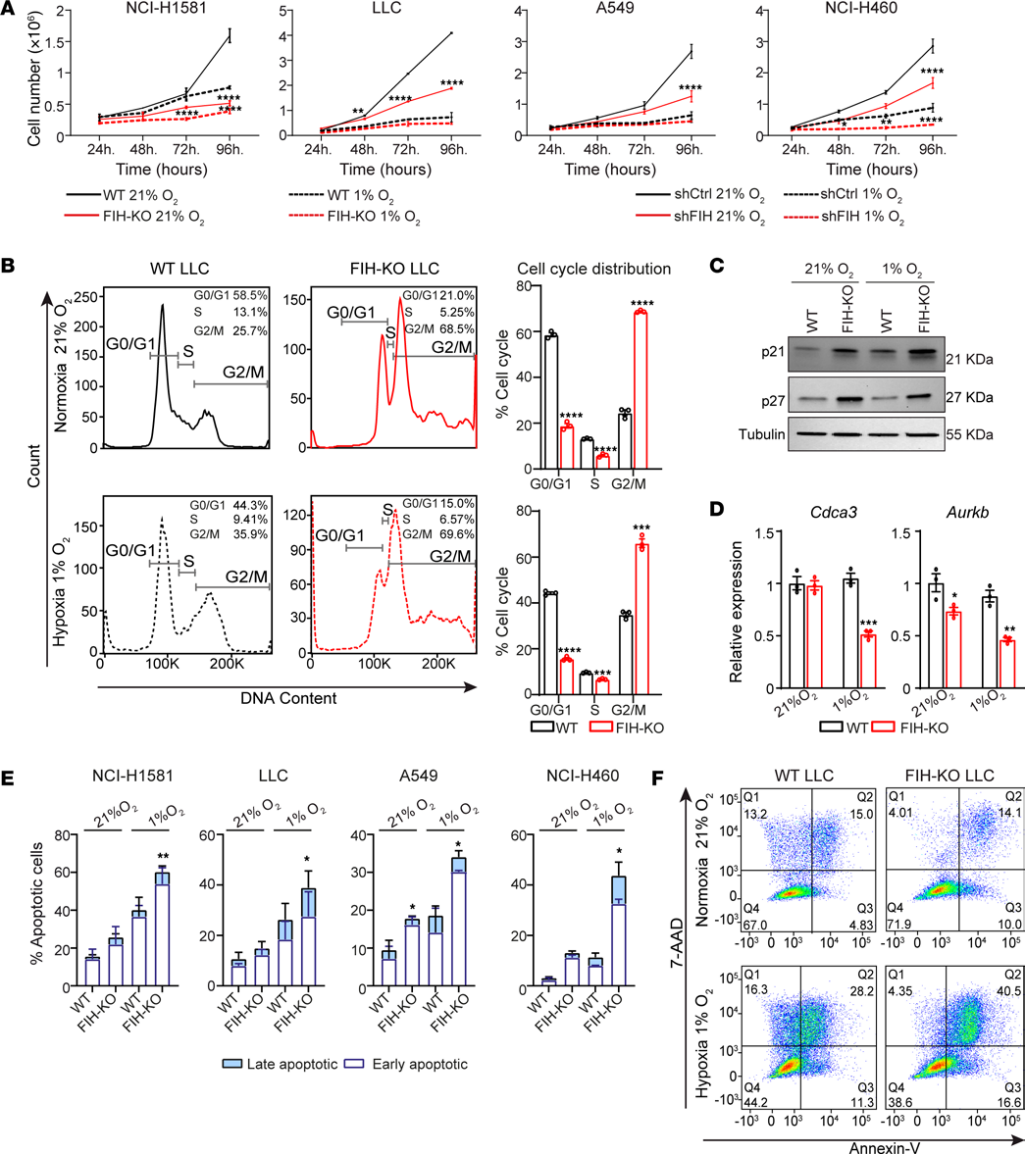
*FIH* deletion in tumor cells impairs cell proliferation and survival in vitro. (**A**) In vitro growth kinetics of WT (black) or *FIH*-KO (red) cells corresponding to the indicated lung cancer cell lines (LLC, NCI-H1581, A549, and NCI-H460) cultured under normoxia (solid line) or hypoxia (dashed line) (*n* = 3, 2-way ANOVA). (**B**) Representative flow cytometry histograms showing cell cycle distribution in WT or *FIH*-KO LLC cells under normoxia (top) or hypoxia (bottom); the percentage of cells in each phase is indicated (left). Bar graphs represent cell cycle phases corresponding to cells cultured under normoxia (top) or hypoxia (bottom) (*n* = 3, unpaired *t* test). (**C**) Representative Western blot showing the expression of p21, p27, and tubulin in WT and *FIH*-KO LLC cells, cultured under normoxia or hypoxia for 16 hours. (**D**) Relative RNA expression levels of *Cdca3* and *Aurkb* genes in WT and *FIH*-KO LLC cells after 16 hours of culture under normoxia or hypoxia, measured by qPCR (*n* = 3, unpaired *t* test). (**E**) Percentage of early (annexin V^+^, 7-AAD^–^) and late (annexin V^+^, 7-AAD^+^) apoptotic cells corresponding to the indicated lung cancer cell lines cultured under normoxia or hypoxia for 48 hours, stained with 7-AAD and annexin V, and measured by flow cytometry (*n* = 3, unpaired *t* test). (**F**) Representative flow cytometry dot plots corresponding to WT or *FIH*-KO LLC cells stained with 7-AAD and annexin V. Data are shown as mean ± SEM; values in each quadrant represent the percentage of cells. *P ≤ 0.05, **P ≤ 0.01, ***P ≤ 0.001, and ****P ≤ 0.0001.

We evaluated the effect of *FIH* deletion in tumor cell proliferation in vitro. Deletion of *FIH* resulted in a reduction of the proliferative capacity of lung cancer cells under both normoxia and hypoxia compared with WT cells (Figure 2A). We investigated whether deletion of *FIH* affected the cell cycle, as a potential driver of the observed reduction in cell proliferation. *FIH* deletion resulted in a higher number of LLC cells arrested at G2/M phase (Figure 2B) and accumulation of cell cycle inhibitors p21 and p27 (Figure 2C). We also found a dysregulation in the expression of key regulators of the cell cycle such as *Cdca3* and *Aurkb* (Figure 2D). Furthermore, *FIH* deletion caused a defect in cell survival, a phenotype that was exacerbated under hypoxia in terms of early and late apoptotic rates (Figure 2, E and F, and Supplemental Figure 6, A–C). In addition, we engineered LLC cells to overexpress *FIH*, without observing an effect on the proliferation rate (Supplemental Figure 7, A and B). Together, our data show that *FIH* deletion suppresses cell proliferation by inducing an arrest in the G2/M-phase and promotes apoptosis in lung cancer cells.

### FIH deletion in lung cancer cells results in changes at the transcriptional level.

**Figure 3:**
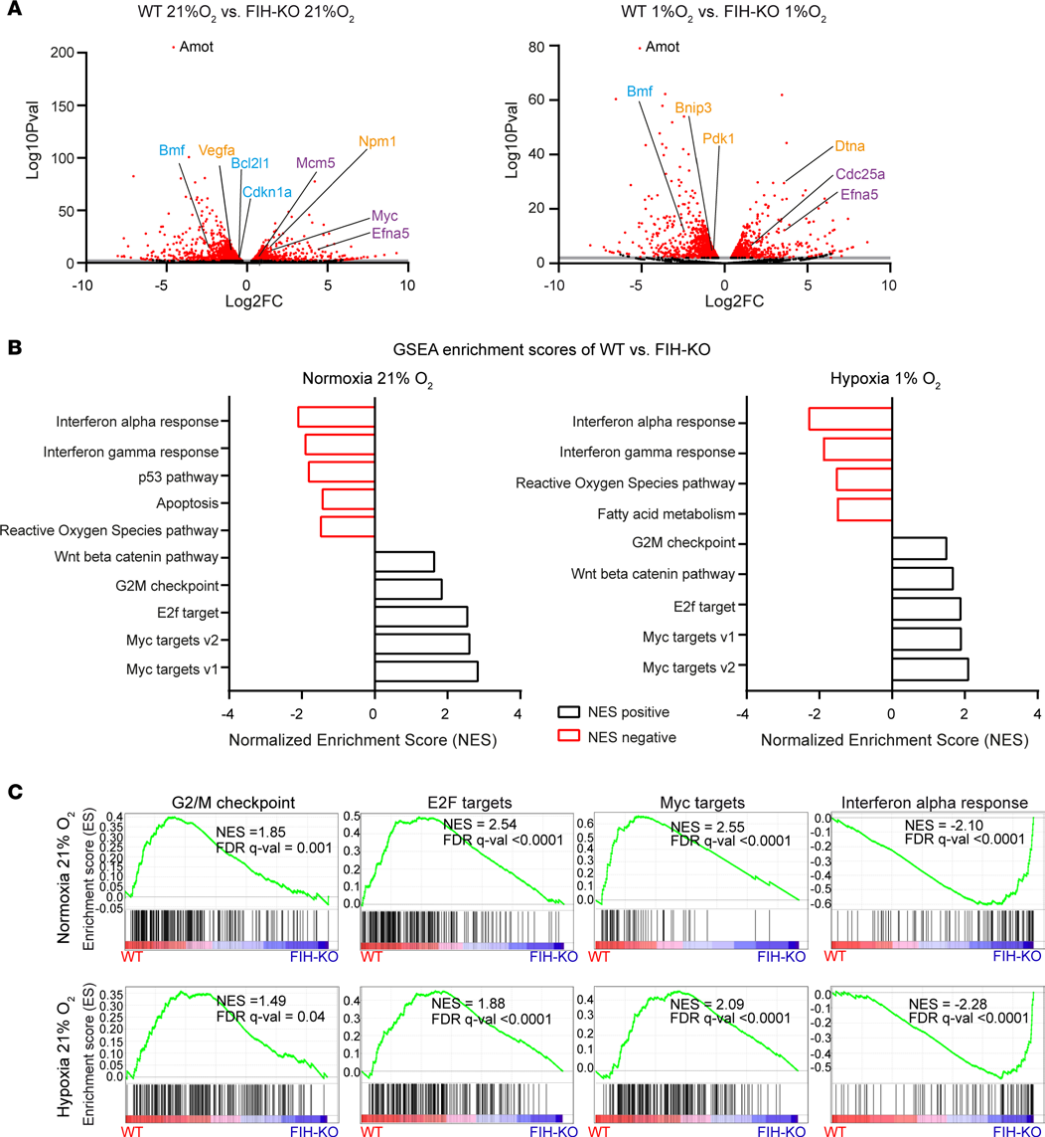
RNA-Seq analysis reveals transcriptional changes in lung cancer cells upon *FIH* deletion. (**A**) Volcano plots showing genes that are differentially expressed in *FIH*-KO LLC cells compared with WT LLC cells. Red dots represent significantly dysregulated genes (*P* < 0.05) that have log_2_ fold changes > 2. Genes corresponding to specific pathways are colored (yellow, hypoxia/cellular response to stress; purple, cell cycle progression; blue, apoptosis; black, Hippo signaling pathway). (**B**) Gene set enrichment analysis (GSEA) showing a selection of significantly dysregulated pathways (*P* < 0.05) in WT versus *FIH*-KO LLC cells cultured under normoxia (right) or hypoxia (left) for 16 hours. Genes were ranked by fold changes and tested for enrichment using the MSigDB hallmark gene sets. (**C**) Individual gene set enrichment plots of selected GSEA hallmarks (G2/M checkpoint, E2F targets, Myc targets and IFN-α response pathway) in WT LLC cells in comparison with *FIH*-KO LLC cells in normoxia (top) or hypoxia (bottom). Normalized enrichment scores (NES) and FDR *q* values are shown.

**Figure 4:**
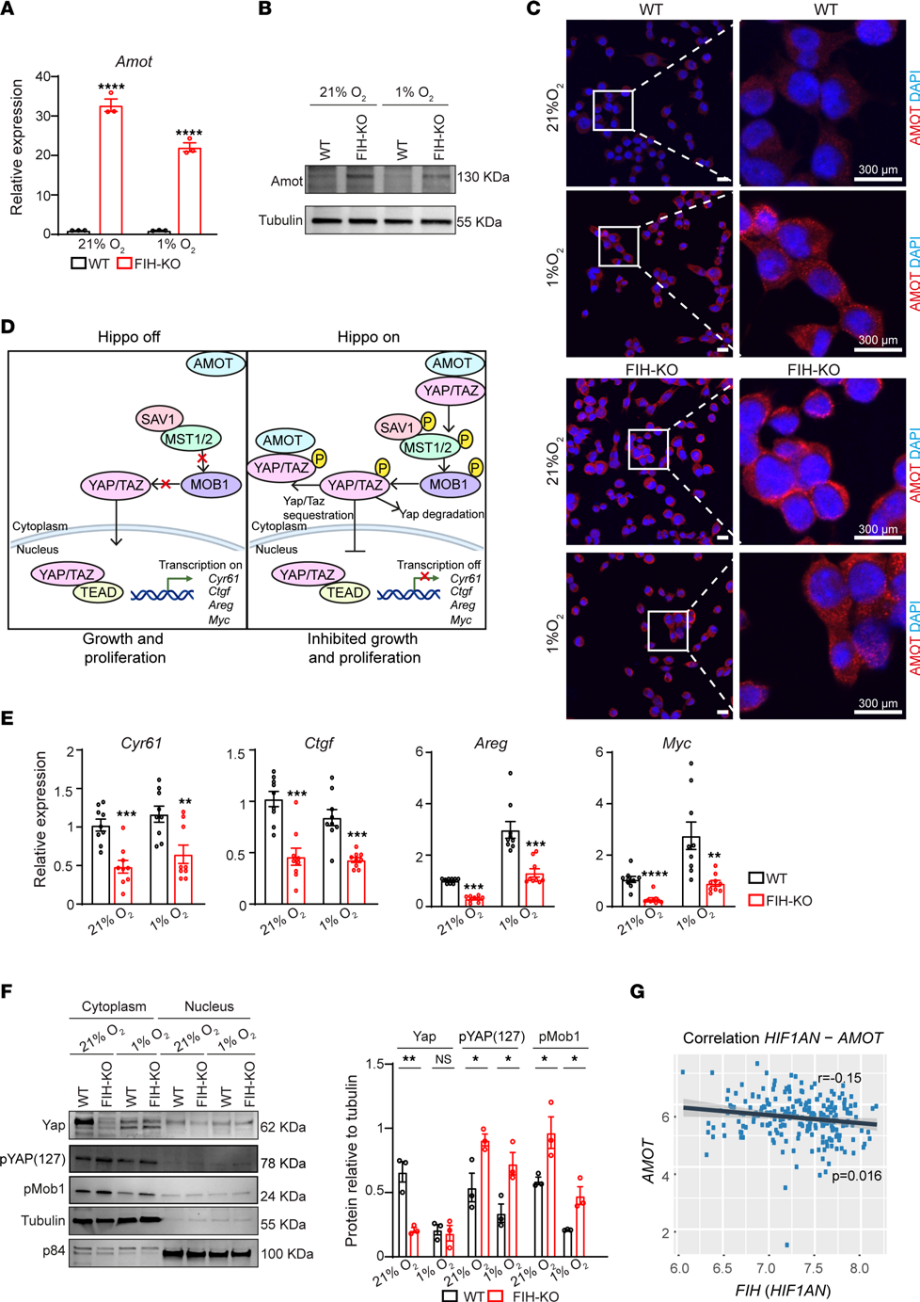
FIH promotes YAP/TAZ signaling in lung cancer cells. (**A**) Relative RNA expression of *Amot* in WT or *FIH*-KO LLC cells under normoxia or hypoxia, measured by qPCR (*n* = 3, unpaired *t* test). (**B**) Immunoblotting showing Amot and tubulin protein levels in WT or *FIH*-KO LLC cells cultured under normoxia or hypoxia. (**C**) IF showing Amot expression in WT or *FIH*-KO LLC cells cultured under normoxia or hypoxia. Enlarged images of WT (top) or *FIH*-KO (bottom) LLC cells are shown in the insets. Scale bars: 300 μm. (**D**) Schematic representation of the Hippo signaling pathway. In the absence of phosphorylation, nuclear translocation of YAP/TAZ acts as a transcriptional coactivator (Hippo pathway off, left). Diverse upstream events, including Amot signaling, can result in YAP phosphorylation, preventing nuclear translocation (Hippo pathway on, right). (**E**) Relative RNA expression of *Cyr61*, *Ctgf*, *Areg*, and *Myc* genes in WT or *FIH*-KO LLC cells cultured under normoxia or hypoxia conditions, measured by qPCR (*n* = 3, unpaired *t* test, technical replicates are shown). (**F**) Western blot showing Yap, phospho-YAP (Ser127), pMob1, tubulin, and nuclear matrix protein p84 (NP84) in WT or *FIH*-KO LLC cells cultured under normoxia or hypoxia (left). Bar graph corresponding to the densitometric quantification of the cytoplasmic fractions of the indicated proteins (right, *n* = 3, unpaired *t* test). (**G**) Correlation between the expression of *FIH* (*HIF1AN*) and *AMOT* mRNA expression in lung cancers (*n* = 246 patients). Data and analysis obtained from Cancertool (Okayama dataset) (81). Data are shown as mean ± SEM. *P ≤ 0.05, **P ≤ 0.01, ***P ≤ 0.001, and ****P ≤ 0.0001. Experiments were performed for 16 hours in each condition indicated.

We next investigated transcriptional changes governed by FIH by performing an RNA-Seq study in WT and *FIH*-KO LLC cells, cultured under normoxia or hypoxia. Volcano plots presented in Figure 3A show genes with expression levels that differ in the absence of *FIH* and are related to potentially relevant pathways such as apoptosis (*Bcl2l1* and *Bmf*), cell cycle progression (*Efna5*, *Myc,* and *Cdc25a*), cellular response to stress or hypoxia (*Dtna*, *Bnip3*, *Npm1*, and *Vegfa*), and the Hippo signaling pathway (*Amot*). Gene set enrichment analysis (GSEA) confirmed the main hallmark pathways that are altered in *FIH*-KO LLC cells compared with WT cells (Supplemental Table 1), including apoptosis, the p53 pathway, cell cycle progression, and the response to reactive oxygen species (ROS), among others (Figure 3, B and C, and Supplemental Figure 8A). Given these findings, we investigated the levels of mitochondrial ROS in lung cancer cells, which are produced as a result of mitochondrial respiration. We found increased ROS levels in *FIH*-KO LLC cells under normoxia or hypoxia conditions compared with WT cells (Supplemental Figure 8B). Interestingly, the RNA-Seq analysis identified genes that are strongly upregulated in the absence of *FIH* under both normoxia and hypoxia (Supplemental Tables 2 and 3), including angiomotin (*Amot*), a known regulator of cell migration and proliferation in lung cancer (65). We validated the observed upregulation of *Amot* in independent experiments performed by quantitative PCR (qPCR) (Figure 4A), immunoblotting (Figure 4B), and IF (Figure 4C), demonstrating that Amot increases upon *FIH* deletion. We identified hypoxia response elements (HREs) in the *Amot* promoter (Supplemental Figure 9A), and both hypoxia and *FIH* deletion resulted in increased HIF binding to this region, as demonstrated by chromatin immunoprecipitation (Supplemental Figure 9B). Amot is a key component of the Hippo signaling pathway and interacts with Yes-associated protein (YAP) transcription coactivator regulating its cellular localization. We hypothesized that *Amot* upregulation is preventing nuclear translocation of YAP, inhibiting oncogenic activity of YAP/TAZ (Figure 4D). In this context, we interrogated the expression of YAP/TAZ transcriptional targets in *FIH*-KO and WT cells, demonstrating that loss of *FIH* results in significantly decreased levels of *Cyr61*, *Ctgf*, *Areg*, and *Myc* (Figure 4E). These findings were reproduced in a human lung cancer cell line (NCI-H1581) that, in the absence of *FIH*, presented higher levels of AMOT and decreased transcription of YAP/TAZ target genes (Supplemental Figure 10, A and B). Furthermore, we compared the protein expression levels and the subcellular localization of YAP in WT and *FIH*-KO LLC cell lines, showing that *FIH* deletion prevents YAP activity and alters the phosphorylation of Mob1 and YAP, key components of the Hippo pathway (Figure 4F). Furthermore, to confirm the negative correlation between *AMOT* and *FIH* expression levels, we interrogated a lung cancer genomic database, finding a moderate but significant association (Figure 4G).

Together, these results suggest that *FIH* deletion promotes Amot accumulation, which in turn acts as a tumor suppressor in lung cancer, acting through the inhibition of downstream Hippo-YAP/TAZ signaling.

### Amot deletion rescues cell proliferation in FIH-KO lung cancer cells.

**Figure 5:**
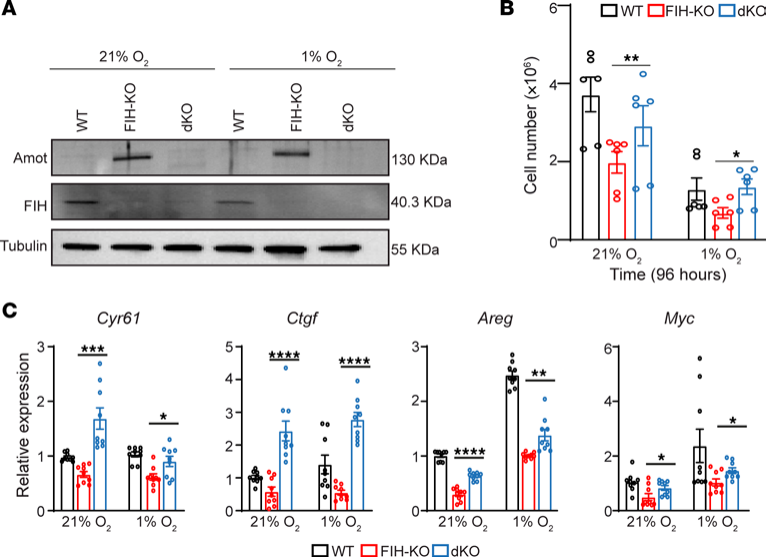
*Amot* deletion rescues the impairment in proliferation of *FIH*-deficient lung cancer cells. (**A**) Western blot showing the levels of Amot, FIH, and tubulin in WT, *FIH*-KO, or double *FIH* and *AMOT* KO (dKO) LLC cells, cultured under normoxia or hypoxia conditions for 16 hours. (**B**) Cell numbers of WT (black), *FIH*-KO (red), or dKO (blue) LLC cells cultured under normoxia or hypoxia as indicated (*n* = 3, unpaired *t* test, technical replicates are shown). (**C**) Relative RNA expression of *Cyr61*, *Ctgf*, *Areg*, and *Myc* genes in WT (black), *FIH*-KO (red), or dKO (blue) LLC cells cultured under normoxia or hypoxia for 16 hours, measured by qPCR (*n* = 3, unpaired *t* test, technical replicates are shown). Data are presented as mean ± SEM. *P ≤ 0.05, **P ≤ 0.01, ***P ≤ 0.001, and ****P ≤ 0.0001.

We evaluated whether the observed decrease in cell proliferation in *FIH*-KO lung cancer cells was a direct result of the accumulation of Amot. We carried out rescue experiments employing LLC cells lacking both *FIH* and *Amot* (double KO; Figure 5A). This approach revealed that the suppression of *Amot* in *FIH*-KO lung cancer cells reinstates the proliferative capacity of the *FIH*-deficient cells (Figure 5B). Furthermore, we observed that, upon *Amot* deletion, *FIH*-KO cells exhibited a rescue of the expression levels of YAP/TAZ transcriptional targets, including *Cyr61*, *Ctgf*, *Areg*, and *Myc* (Figure 5C). Our findings substantiate that *Amot* acts as a suppressor of cell proliferation in *FIH*-KO cells.

### FIH deletion in tumor cells results in reduced tumor growth and survival in vivo.

**Figure 6:**
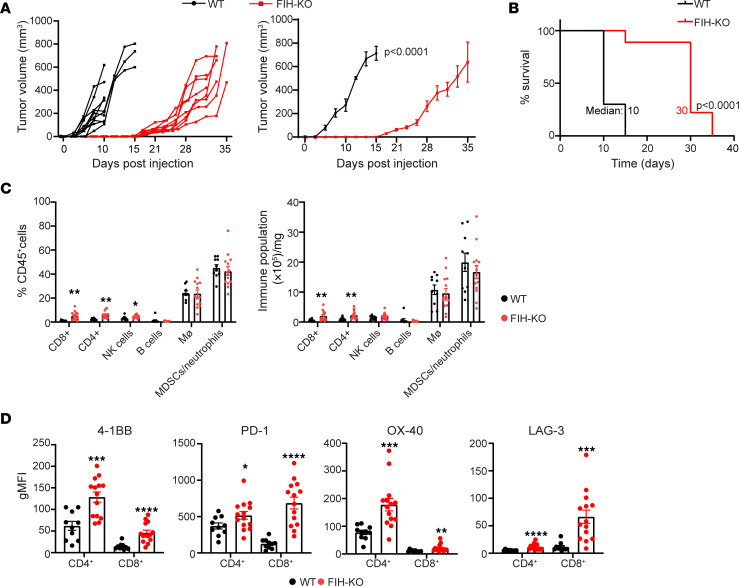
*FIH* deletion inhibits lung cancer cell growth in vivo. (**A**) Individual tumor development of WT (black) or *FIH*-KO (red) LLC tumors are indicated (left). Tumor growth was measured every other day, and Data are shown as mean ± SEM at the indicated time points (right). Statistical test represents 2-way repeated-measures ANOVA in days 8, 10, 13, 14, and 15. (**B**) Kaplan-Meier survival analysis of the indicated groups. Mice were euthanized when tumors reached 12 mm. Statistical analysis for survival curves represents a log-rank test. Values represent median survival days. For **A** and **B**, data from 1 representative experiment is shown (*n* = 3 independent experiments). *n* = 9–10 mice per group. (**C**) The percentages (left) and absolute numbers (right) of immune populations in tumors within live CD45^+^ cells were analyzed by flow cytometry. (**D**) gMFI values corresponding to the level of expression of 4-1BB, PD-1, OX-40, and LAG-3 were determined on tumor-infiltrating CD4^+^ and CD8^+^ T cells by flow cytometry. A pool of 2 independent experiments is represented in **C** and **D**. *n* = 10–14 mice per group. *P* values were calculated by Mann Whitney *U* nonparametric test. Data are presented as mean ± SEM. *P ≤ 0.05, **P ≤ 0.01, ***P ≤ 0.001, and ****P ≤ 0.0001.

**Figure 7:**
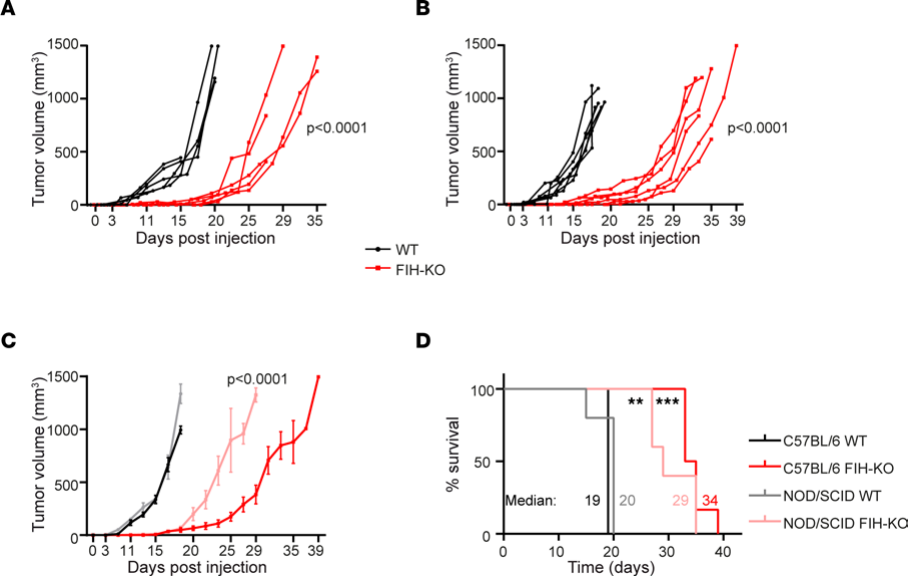
*FIH* deletion inhibits lung cancer cell growth in vivo in immunodeficient mice. (**A**) Tumor growth of WT (black) or *FIH*-KO (red) LLC tumors in immunocompromised NOD/SCID mice; individual tumor development is indicated. *n* = 5 mice per group. (**B**) Tumor growth of WT (black) or *FIH*-KO (red) LLC tumors (left) in immunocompetent C57BL/6 mice; individual tumor development are indicated. *n* = 6 mice per group. (**C**) Tumor growth of WT or *FIH*-KO LLC tumors growing in NOD/SCID (**A**) or C57BL/6 (**B**) mice. Data are shown as mean ± SEM. Statistical test represents 2-way repeated-measures ANOVAs in days 22, 25, 27, and 29. *n* = 5–6 mice per group. (**D**) Kaplan-Meier survival analysis corresponding to the indicated groups. Mice were euthanized when tumors reached 15 mm. Statistical analysis of survival curves represents log-rank tests. Indicated values represent the median survival for each group. *n* = 5–6 per group. Data are presented as mean ± SEM. **P ≤ 0.01 and ***P ≤ 0.001.

We next investigated whether the absence of *FIH* could affect tumor growth. To this end, we s.c. injected WT or *FIH*-KO LLC cells in C57BL/6 mice. *FIH* deletion in lung tumor cells led to a significant decrease in the rate of tumor growth (Figure 6A), which in turn had a positive effect on the survival rate (Figure 6B). In addition, we analyzed the composition of the immune infiltrate in WT and *FIH*-KO LLC tumors (Figure 6C) and spleens. While the spleen composition in both experimental groups remained unchanged (data not shown), the immune infiltrate of *FIH*-KO tumors was characterized by an increase in terms of percentage and absolute numbers of tumor-infiltrating lymphocytes (CD4^+^, CD8^+^, and NKs) compared with WT tumors (Figure 6C). Importantly, we found that CD4^+^ and CD8^+^ T cells infiltrating *FIH*-KO tumors expressed higher levels of the costimulatory (4-1BB and OX40) and coinhibitory (PD-1 and LAG-3) markers (Figure 6D). In view of this, we next determined if the observed reduction in tumor growth in the absence of *FIH* was dependent on antitumor immunity. To assess this possibility, WT or *FIH*-KO LLC cells were injected into NOD/SCID immunodeficient female mice, resulting in a reduction in tumor growth in the absence of *FIH* (Figure 7, A–D). Therefore, these results demonstrate that *FIH* deletion reduced tumor growth through an intrinsic mechanism.

### FIH promotes lung metastasis in vivo.

**Figure 8:**
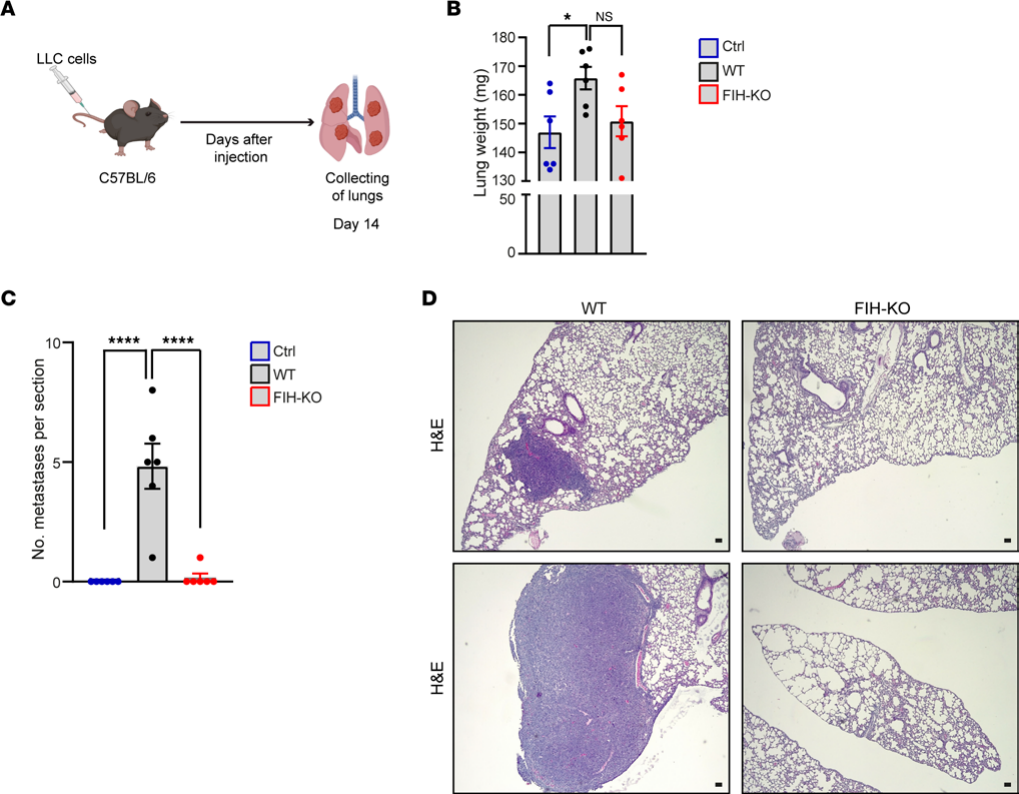
FIH promotes lung metastasis in vivo. (**A**) Schematic representation of the workflow to assess metastatic success upon *FIH* deletion. LLC cells were i.v. injected in C57BL/6 mice and lungs were collected on day 14. (**B**) Lung weights of control mice (blue) or mice receiving WT (black) or *FIH*-KO (red) tumor cells are shown (*n* = 6, 1-way ANOVA). (**C**) Number of metastases per lung section were quantified after staining with H&E (*n* = 6, 1-way ANOVA). (**D**) Representative images of lung histology are shown; sections were stained with H&E. Scale bars: 300 μm. Data are shown as mean ± SEM. *P ≤ 0.05 and ****P ≤ 0.0001.

We next characterized the lung metastatic capacity of *FIH*-KO lung cancer cell lines injected i.v. in immunocompetent female mice and examined metastatic colonization of the lung by histology (Figure 8A). Interestingly, *FIH* deletion prevented lung colonization, resulting in lower total lung weight in mice injected with *FIH*-KO cells compared with WT controls (Figure 8B). Remarkably, metastatic lesions were observed in the experimental group receiving WT cells; they formed numerous and large metastatic foci compared with *FIH*-deficient LLC cell lines (Figures 8, C and D). Therefore, these findings show that FIH favors the metastatic process.

### FIH expression correlates with poor lung cancer patient survival.

**Figure 9:**
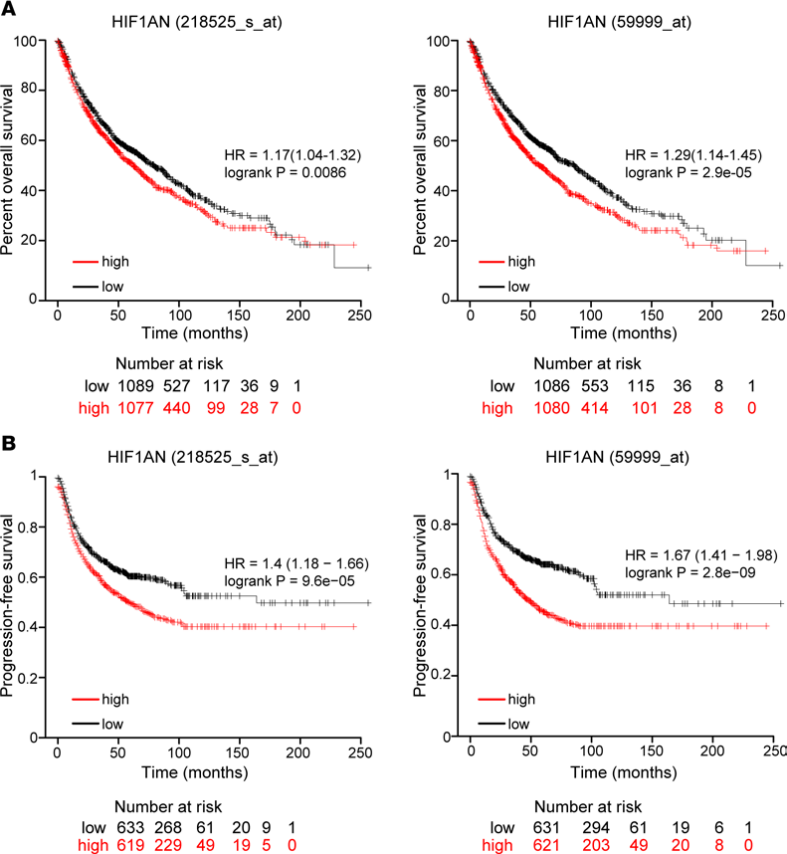
High FIH expression correlates with poor survival in patients with NSCLC. (**A**) Kaplan-Meier overall survival plot of patients with NSCLC (*n* = 2166), classified by the level of FIH expression: high (red) or low (black), measured with Affymetrix arrays (IDs: 218525_s_at [right] and 59999_at [left]). A log-rank test for each panel was performed, and *P* values are indicated. (**B**) Kaplan-Meier shows progression-free survival of patients with lung cancer (*n* = 1252) classified by their level of FIH expression: high (red) and low (black).

We finally explored whether different expression levels of FIH had an effect on the prognosis of patients with lung cancer. Kaplan-Meier curves shown on Figure 9A and Supplemental Figure 11A show that a high expression of FIH correlates with a worse overall survival in patients with lung cancer. Furthermore, patients with high FIH expression showed significantly worse progression-free survival (Figure 9B and Supplemental Figure 11B).

## Discussion

HIFs are considered factors that facilitate tumor growth and progression, given their central role in the adaptation of cancer cells to limited O_2_ availability in the TME (8, 66, 67). However, several studies focusing on the analysis of expression of HIF hydroxylases demonstrate a correlation between high levels of PHDs or FIH and a worse survival in some tumor types (58, 62, 68, 69). These studies do not provide a conclusive explanation of the mechanism underlying this paradox. In this context, the role of hydroxylases that control HIF accumulation and activity in cancer is not well understood.

FIH is ubiquitously expressed in mammals, with varying degrees of expression across tissues. While hypoxia induces the expression of PHD2 (70), the level of FIH remains unchanged. Here we demonstrate that FIH is expressed in normal lung and both LUAD and LUSC, with a predominant cytoplasmic localization. Given that the role of FIH in lung cancer is largely unexplored, we generated various genetic cell models that demonstrated that FIH controls metabolism, proliferation, and survival of lung cancer cells. The cellular metabolic phenotype of *FIH*-KO cells was more evident under hypoxia and consistent with increased HIF activity, resulting in increased glycolytic rate as reported in studies carried out with *FIH*-deficient transgenic mice (45, 48). These results indicate that FIH is a functional regulator of the hypoxia response in lung cancer. Interestingly, this effect is also accompanied by an increase in the expression of PHD2 and a subsequent decrease in the total protein levels of HIF1A, suggesting that asparaginyl hydroxylation could also modulate the hydroxylation of prolyl residues on HIF, driving its degradation (44).

We demonstrate that FIH promotes lung cancer cell proliferation and survival, acting on the cell cycle. This finding is relevant from the mechanistic perspective, given that HIF activity is often associated with tumor progression and malignancy in lung cancer but can also induce cell cycle arrest (71, 72). Similar observations have been described in other tumor types such as renal cancer (50), colon adenocarcinoma, and melanoma (53), indicating that these effects extend into other solid tumor types beyond lung cancer.

To further understand the molecular mechanisms underlying the observed effect on lung cancer progression, we carried out transcriptomic analyses revealing that FIH deletion results in the dysregulation of several key pathways that control cell proliferation and survival, including the p53/p21 axis (53). Further studies are required to ascertain if HIFs or other known FIH substrates, such as ARD-containing proteins or OTUB1, contribute to the cell proliferative capacity. Interestingly, FIH deletion resulted in a dramatic increase in the expression of Amot, a member of the Hippo pathway that contains functional HREs in its promoter. Exposure of cells to hypoxia alone does not drive the expression of Amot to the level observed upon FIH deletion, indicating that other HIF-independent mechanisms could be responsible of Amot accumulation. Amot controls YAP/TAZ signaling and acts as a tumor suppressor and regulator of migration in lung cancer (65). In this context, we show that *FIH* deletion leads to reduced downstream YAP/TAZ signaling.

Pharmacologic modulation of HIF activity regulates key hallmarks of the hypoxia pathway, resulting in clinical benefit in some disease segments. For instance, PHD inhibitors, including roxadustat and vadadustat, are being used in the clinic for the treatment of renal anemia (73). At the preclinical level, a few studies have explored the potential use of PHD inhibitors in the treatment of cancer, demonstrating efficacy in lung and breast cancer models (74, 75). Our data show that FIH-deficient tumors present reduced growth and are infiltrated by higher numbers of effector T cells. In this context, we hypothesize that metabolic adaptation of *FIH*-deficient tumor cells influences immune responses through the accumulation of byproducts of HIF metabolism, such as lactate, that can be directly uptaken by T cells promoting effector activity (76). Therefore, pharmacologic inhibition of FIH (77, 78) could offer a differentiated strategy to control tumor growth and prevent metastasis. A specific inhibitor of FIH would act on tumor cells to restrict proliferation and survival, offering opportunities for combinations with other therapies such as immunotherapeutic agents targeting the PD-1/PD-L1 axis.

In summary, we demonstrate that FIH contributed to lung cancer progression and metastasis, suggesting that FIH inhibition could be a therapeutic strategy.

## Methods

### Mice.

C57BL/6 and NSG (NOD.Cg-Prkdc^scid^Il2rg^tm1Wjl^/SzJ) female mice (6–10 weeks old) were acquired from Charles River Laboratory. Animal procedures were performed following the ethical guidelines established by the Biosafety and Welfare Committee at CIC bioGUNE and the recommendations from Association for Assessment and Accreditation of Laboratory Animal Care International (AAALAC). Mice were housed in individually ventilated cages in a pathogen-free conditions at CIC bioGUNE Animal Facility.

### In vivo tumor growth.

A total of 5 ***×*** 10^5^ WT or *FIH*-KO murine LLC cells were injected s.c. in the right flank of 6- to 10-week-old female C57BL/6 or NSG mice in 100 μL of PBS. Mice were monitored for tumor growth every other day using a digital caliper. Tumor sizes were measured, and tumor volumes were calculated using the formula: V (mm^3^) = (π ***×*** L ***×*** W^2^)/6, where L is tumor length and W is tumor width. Mice were sacrificed when L reached between 12 and 15 mm.

### Cell lines and cell culture.

The murine LLC (LLC1) cell line was obtained from the American Type Culture Collection (ATCC, catalog CRL-1642) and cultured in DMEM (Thermo Fisher Scientific, catalog 41966029) according to standard mammalian tissue culture protocols and sterile techniques. The NCI-H460 human lung epithelial carcinoma cell line was obtained from DSMZ (German Collection of Microorganisms and Cell Lines, catalog ACC737) and cultured in RPMI medium 1640 GlutaMAX (Thermo Fisher Scientific, catalog 61870010). The A549 human lung epithelial carcinoma cell line (ATCC, catalog CCL-185) was cultured in DMEM F-12K (Thermo Fisher Scientific, catalog 31331028). The NCI-H1581 human lung epithelial NSCLC stage 4 cell line (ATCC, catalog CRL-5878) was cultured in RPMI medium 1640 GlutaMAX (Thermo Fisher Scientific, catalog 61870010). HEK 293 cells (Takara Bio Inc., catalog 632180) were cultured in DMEM (Thermo Fisher Scientific, catalog 41966029). All cell lines were cultured in media supplemented with 10% FBS (Thermo Fisher Scientific, catalog 10270106) and 1% penicillin-streptomycin (Thermo Fisher Scientific, catalog 15140122) and tested for mycoplasma using MycoAlert mycoplasma detection kit (Lonza, catalog LT27-221) following the manufacturer’s instructions. Cells were maintained at 37°C in a 5% CO_2_ atmosphere. Hypoxia cultures were performed at 1% O_2_, and other different levels of O_2_ — 10% O_2_, 5% O_2_, and 2.5% O_2_ — in an InVivo2 400 hypoxia station (Ruskinn Technologies). To measure cell proliferation in vitro, cells were cultured in a 5% CO_2_ and 21% O_2_ or 1% O_2_ atmosphere at 37°C for 4 days, stained with trypan blue, and counted in an automated cell counter (Countess Automated Cell Counter 3, Thermo Fisher Scientific).

### KO of FIH by CRISPR/Cas9.

Deletion of *FIH* in mouse LLC cells was performed using the following TrueGuide Synthetic gRNAs (sgRNAs) from Thermo Fisher Scientific: CRISPR425134_SGM (target DNA sequence: 5’-TAAGCCAAGGTCCAACAGGG-3’, catalog A35511) targeting the exon 2 of mouse *Hif1an* gene. Deletion of *FIH* in human NCI-H1581 cells was performed using the following TrueGuide Synthetic gRNA from Thermo Fisher Scientific: CRISPR1082241_SGM (target DNA sequence: 5’-GGAAGCTATAACTGCGCAAC-3’, catalog A35533) targeting the exon 1 of the human *HIF1AN* gene. Ribonucleoprotein (RNP) complexes were generated with 37.5 pmol of TrueCut Cas9 protein v2 (Invitrogen, catalog A36496) and 37.5 pmol of each sgRNA. RNP complexes were introduced into cells using the Lipofectamine CRISPRMAX Cas9 Transfection reagent (Invitrogen, catalog CMAX00001) and Opti-MEM reduced serum medium (Thermo Fisher Scientific, catalog 31985062) following instructions by Invitrogen. A nontargeting sgRNA was used as a negative control (Invitrogen, catalog A35526), and a mouse Rosa26 sgRNA was used as a positive control (Invitrogen, catalog A35525). GeneArt Genomic Cleavage Detection Kit (Thermo Fisher Scientific, catalog A24372) was used to verify gene editing efficiency in a pooled cell population transfected with the TrueGuide Positive Control following the protocol by Invitrogen. Seventy-two hours after transfection, single-cell dilutions were performed in 96-well plates. The absence of FIH was confirmed by Western blot, and selected clones were sequenced by Sanger DNA sequencing (StabVida or EUROFINS) using specific primers (Supplemental Table 4) flanking the sgRNA target region.

### Knockdown of FIH by RNA interference.

Lung carcinoma cells (A549 and NCI-H460 cell lines) were transduced with lentiviral particles carrying short hairpin RNA (shRNA) against human *HIF1AN* (RefSeq accession no. NM_017902.3; https://www.ncbi.nlm.nih.gov/nuccore/NM_017902.3) (Supplemental Table 4). Inserts were prepared by annealing shRNA oligonucleotides containing 2 complementary target sequences linked by a short loop and then were cloned into the BamHI/EcoRI sites of the shRNA lentiviral vector pGreenPuro-shRNA-Stx3S-C4 (Supplemental Table 5) according to the manufacturer’s instructions. The resulting constructs were sequenced using specific primers (Supplemental Table 4).

### Lentiviral plasmid construction.

Deletion of *Amot* in mouse *FIH*-KO LLC cells was performed using the following TrueGuide Synthetic gRNA (sgRNA) from Thermo Fisher Scientific: CRISPR469888_SGM (target DNA sequence: 5’-CCAGTCAGAAACGGCGTCA-3’, catalog A35533) that was synthetized and subcloned into a LentiCRISPRv2 vector (GenScript). For FIH overexpression, the codon-optimized sequence of mouse full-length FIH was synthetized and subcloned into a puro pLV-MSCV lentiviral vector (GenScript) and transduced into LLC cells with lentiviral particles.

For the generation of lentiviral particles, confluent HEK 293T cells (50%–70%) were transfected with JetPEI kit (Polyplus transfection, catalog 101-10N) using pGreenPuro-shRNA-Stx3S-C4 and third-generation plasmids: pRSV-Rev, pMDLg/pRRE, and pMD2.G, as packaging plasmids (Supplemental Table 5) as well as the VSV-G plasmid encoding for the envelope (Supplemental Table 5). Viral particles were harvested from the supernatant 48 hours after transfection and filtered through a 0.45 μm filter (VWR, catalog 514-0063). After puromycin titration, A549, NCI-H460, and LLC cells were infected with lentiviral particles and selected with 1.5 μg puromycin for A549 and NCI-H460 cells and 2 μg puromycin for LLC cells.

### Flow cytometry.

Mouse tumor samples were minced finely with a scalpel blade in a Petri dish and incubated for 30 minutes at 37°C with type I collagenase (0.5 mg/mL) (Sigma-Aldrich, catalog C0130) in DMEM. After digestion, tissue and spleen samples were filtered through a 70 μm cell strainer (BD Biosciences) to obtain single-cell suspensions, followed by RBC lysis performed with ACK buffer (Thermo Fisher Scientific, catalog A1049201) for 3 minutes at room temperature. Samples were stained with the LIVE/DEAD fixable blue or green dead cell stain kit (Thermo Fisher Scientific, catalogs L34962 and L34970, respectively) (1:1,000 in PBS) for 30 minutes at 4°C protected from light. Cells were washed in PBS and incubated with fluorochrome-conjugated antibodies, listed in Supplemental Table 6, in flow cytometry staining buffer (Invitrogen, catalog 00-4222-26) for 30 minutes at 4°C in the dark. Cells were blocked prior to antibody staining with a TruStain FcX blocker (BioLegend, catalog 101320) (1:50 in staining buffer) for 10 minutes at room temperature. Finally, cells were washed once in staining buffer, stained with CountBright absolute counting beads (Thermo Fisher Scientific, catalog C36995) to determine absolute cell number, and acquired in a flow cytometer (BD Biosciences). All centrifugation steps were performed at 450*g* for 5 minutes at 4°C.

For cell cycle analysis, cells were harvested, washed in PBS (Thermo Fisher Scientific, Fisher BioReagent, catalog BP3994), and fixed with ice-cold 70% ethanol for 30 minutes at 4°C. Cell pellets were washed twice in PBS and resuspended in 1 mg/mL DAPI (Invitrogen, catalog D1306). To measure apoptosis, cells were cultured for 24 hours under normoxia or hypoxia, harvested, washed twice with ice-cold staining buffer, and stained with BV421-annexin V and 7-AAD using the Apoptosis Detection Kit with 7-AAD (BioLegend, catalog 640922). To quantify ROS, cells were cultured for 48 hours, stained with MitoSOX Red (5 μM) (Thermo Fisher Scientific, catalog M36007), and acquired in a flow cytometer after LIVE/DEAD staining. All samples were acquired in a BD FACSymphony flow cytometer (BD Biosciences). Data were analyzed using FlowJo version 10 (BD Biosciences).

### Western blot.

Total cell lysates were collected using RIPA buffer (Thermo Fisher Scientific, catalog 89900). Nuclear and cytoplasmic protein extracts were isolated using NE-PER Nuclear and Cytoplasmic Extraction Kit (Thermo Fisher Scientific, catalog 78835). Protein quantification was performed using the Pierce BCA Protein Assay Kit (Thermo Fisher Scientific, catalog 23227). Samples were mixed with LDS sample buffer (Invitrogen, catalog NP0007) containing DTT (Melford, catalog MB1015), heated for 10 minutes at 95°C, and resolved in a 7.5% or 4%–15% Mini-PROTEAN TGX precast protein gels (Bio-Rad, catalogs 4561023 and 4561085, respectively) with 10***×*** Tris/Glycine/SDS electrophoresis buffer (Bio-Rad, catalog 1704156) in parallel with the PageRuler Plus Prestained Protein Ladder (Thermo Fisher Scientific, catalog 26619). Protein transfer to 0.2 μm PVDF membranes (Bio-Rad, catalog 1704156) was carried out in a Trans-Blot Turbo transfer system (Bio-Rad). Membranes were blocked for 1 hour in 5% skim milk (MilliporeSigma, catalog 70166), diluted in PBS 0.5% Tween-20 (T-PBS, Sigma-Aldrich, catalog 9005-64-5), and incubated overnight with primary antibodies (Supplemental Table 7) and the corresponding secondary HRP-conjugated antibodies: anti–mouse IgG (Cell Signaling Technology, catalog S301677076S) or anti–rabbit IgG (Cell Signaling Technology, catalog S301677074S). Chemiluminescence detection was performed using Clarity Max Western ECL Substrate (Bio-Rad, catalog 170506) in an iBright CL1500 system (Invitrogen). Band intensities were determined by densitometric analysis using ImageJ software (NIH). See complete unedited blots in the supplemental material.

### IF and IHC.

For IF, cells were incubated on coverslips under normoxia and hypoxia for 24 hours, rinsed in PBS, fixed, and permeabilized with ice-cold 100% methanol for 15 minutes at –20°C. Samples were rinsed 3 times in PBS and blocked in 1 mL of PBS 0.1% Triton X-100 3% BSA at room temperature for 30 minutes under agitation. Cells were stained with anti-FIH antibody (1:50, mouse monoclonal antibody, Santa Cruz Biotechnology Inc., catalog SC-271780) and anti-AMOT (1:100, rabbit polyclonal antibody, Thermo Fisher Scientific, catalog THPA5103603). After overnight incubation with primary antibodies, samples were washed 5 times with PBS 0.1% Triton X-100. Protein expression was detected after staining with secondary antibody anti–mouse Alexa Fluor 647 (1:500, Thermo Fisher Scientific, catalog A21235) for 45 minutes at room temperature. DAPI was used to stain nuclei. ProLong Diamond Antifade Mountant (Invitrogen, catalog P36965) was used. Samples were observed using a confocal microscope (Leica SP8 Lightning).

For IHC, paraffin-embedded tissue microarrays were acquired from Novus Biologicals (NBP2-42077) (Supplemental Table 8). Tissue sections were deparaffinized with Histo-Clear II (National Diagnostics, catalog HS-202) and rehydrated in 100%, 95%, and 70% ethanol and distilled water. Antigen retrieval was performed in a steamer filled with sodium citrate buffer at pH 6.0. Endogenous peroxidase activity was quenched for 10 minutes with 3% hydrogen peroxide. Samples were washed with PBS and blocked with normal goat blocking serum (Bio-Techne, catalog MP7451) for 20 minutes at room temperature to reduce nonspecific staining. Samples were then incubated overnight with primary antibody against anti-FIH (1:750, Atlas Antibodies, catalog HPA048742), followed by an anti–rabbit IgG ImmPRESS secondary antibody for 30 minutes (Bio-Techne, catalog MP7451). Liquid diaminobenzidine (DAB, Vector DAB Kit, catalog SK-4105) was used for 10 minutes, and sections were counterstained with Mayer’s hematoxylin (Merck, catalog MHS16). Slides were mounted with DPX Mountant (Merck, catalog 06522). Images were acquired in an AxioImager D1 light microscope (Carl Zeiss).

### qPCR.

Total RNA was extracted using the NucleoSpin RNA kit (Macherey-Nagel, catalog 740955.250). The cDNA was synthesized by reverse transcription (RT)-PCR from 1 μg of purified RNA with M-MLV reverse transcriptase (Thermo Fisher Scientific, catalog 28025-013) and random primers (Thermo Fisher Scientific, catalog 58875). qPCR reactions were performed in a Viia 7 Real-Time PCR System (Thermo Fisher Scientific) using PerfeCTa SYBR Green SuperMix reagent (Quantabio, catalog 95056-500) and gene-specific primers (Supplemental Table 4). Data were analyzed using QuantStudio version 1.3 (Thermo Fisher Scientific). Relative expression was calculated using the 2^–ΔΔCt^ method. The *RPLP0* gene was used as housekeeping gene.

### RNA-Seq library preparation, sequencing, and data analysis.

RNA sample quantity and quality were measured using Qubit RNA HS Assay Kit (Thermo Fisher Scientific, catalog Q32855) and Agilent RNA 6000 Nano Chips (Agilent Technologies, catalog 5067-1511), respectively. The cDNA was synthesized with SuperScript-II Reverse Transcriptase (Thermo Fisher Scientific, catalog 18064-014), and libraries were prepared using “TruSeq Stranded mRNA library Prep” kit (Illumina Inc., catalog 20020594) and TruSeq RNA CD Index Plate (96 Indexes, 96 samples; Illumina Inc., catalog 20019792), following TruSeq Stranded mRNA Sample Preparation Guide (part no. 15031058 Rev. E). The final dsDNA libraries were quantified by Qubit dsDNA HS DNA Kit (Thermo Fisher Scientific, catalog Q32854) and qualified by an Agilent 2100 Bioanalyzer using Agilent High Sensitivity DNA kit (Agilent Technologies, catalog 5067-4626). Paired-end Illumina sequencing was performed.

### ChIP qPCR.

WT or *FIH*-KO LLC cells were grown to 80%–90% confluence in 150 cm culture dishes. The following day, ChIP assay was performed using SimpleCHIP Enzymatic Chromatin IP Kit (Magnetic Beads) (Cell Signaling Technology, catalog 9003) using 10 μg of chromatin and following the manufacturer’s instructions. The following antibodies were used: anti-HIF1A ChIP antibody (Cell Signaling Technology, catalog 36169) or anti–rabbit IgG (Cell Signaling Technology, catalog 2729). Chromatin was immunoprecipitated overnight at 4°C, and HIF1A binding sites were assayed via qPCR using 2 μL of DNA of either input, IgG-IP, or HIF1A-IP per reaction in triplicate. The qPCR oligos are listed in Supplemental Table 4.

### Glycolysis and lactate assays.

Glucose levels in cell supernatants were quantified by a colorimetric glucose detection kit (Thermo Fisher Scientific, catalog EIAGLUC). Cellular glucose uptake was measured using 100 μM of 2-NBDG (Thermo Fisher Scientific, catalog N13195) in glucose-free RPMI or DMEM (Thermo Fisher Scientific, catalog 41966-029) by flow cytometry. Lactate levels in cell supernatants were determined by a colorimetric assay kit (Sigma-Aldrich, catalog MAK064).

### Dual luciferase HRE reporter assay.

WT or *FIH*-KO LLC and NCI-H1581 cells were seeded in 24-well plates at a density of 1 ***×*** 10^5^ cells/well. Next day, cells were cotransfected with HRE luciferase (Supplemental Table 5) and pNK1.1 TK (Promega, catalog N1610) plasmids using Lipofectamine 2000 (Thermo Fisher Scientific, catalog 11668030) according to the manufacturer’s instructions. After transfection, cells were treated with 1 mM DMOG (Merck, catalog D3695) for 16 hours, and the luciferase assays were performed in triplicate using the Dual-Glo Luciferase Assay System (Promega, catalog N1610) and a PerkinElmer Victor Nivo plate reader.

### Bioinformatic analysis.

For the RNA-Seq analysis, sequencing data were converted into raw data (FASTQ files) using the Illumina bcl2fastq Conversion Software. The raw sequence reads were aligned to the Genome Reference Consortium Mouse Reference build number 39 (GRCm39) of the mouse genome (mm39) using STAR2 (79), and read counts for genes were prepared by HTSeq script from which transcripts per million (TPM) were calculated. Differential gene expression analysis was performed by the R package using DESeq2.

### GSEA.

GSEA was performed as described before (80) using publicly available software (https://www.gsea-msigdb.org/gsea/index.jsp). provided by the Broad Institute (RRID:SCR_003199. Version 3.0) with the Hallmarks gene set provided by the Molecular Signature Database (MSigDB; www.broad.mit.edu/gsea). Pathways were considered enriched if their FDR adjusted *P* value was less than 0.05.

### FIH gene expression across human normal and tumor cells.

Data from 30 normal tissues (TCGA GTEX data set) was analyzed with OmicSoft OncoLand (OmicSoft Corp.) and represented as log_2_(FPKM + 0.1). FIH expression in LUAD, LUSC, or healthy participants was analyzed with GEPIA. Normalized raw data was represented as –log_2_(TPM + 1). Expression of *FIH* in 175 different lung cancer cells (CCLE-B37_G33 database) was analyzed with OmicSoft OncoLand (OmicSoft Corp.).

### Correlations between FIH and AMOT.

Spearman correlation analyses between FIH and AMOT was performed in the Okayama study (81) using Cancertool (82).

### Survival analysis.

The overall survival rate and progression-free survival of patients with lung cancer based on the level of FIH (*HIF1AN*) expression were obtained using Kaplan-Meier Plotter (https://kmplot.com/) (83).

### Statistics.

Statistical analyses were performed using GraphPad PRISM 8 software (GraphPad). Plots show mean ± SEM. Unpaired *t* test was used to analyze experiments with 2 groups. For comparison among multiple groups, 1-way ANOVA was used. For comparison of grouped data, a 2-way ANOVA was used. Mann-Whitney *U* test was used to analyze two independent samples. Overall survival and disease-free survival curves were plotted by Kaplan-Meier survival analysis and log-rank test. Significant *P* values are indicated by *P ≤ 0.05, **P ≤ 0.01, ***P ≤ 0.001 and ****P ≤ 0.0001.

### Study approval.

Animal procedures were performed in compliance with the ethical guidelines established at CIC bioGUNE by the Biosafety and Welfare Committee and the AAALAC. All procedures relating to animals were approved by Diputación Foral Vizcaya under the project no. P-CBG-CBBA-1022.

### Data availability.

RNA-Seq data sets generated in this publication are available in GEO database with the series accession no. GSE221505. Values for all data points in graphs are reported in the Supporting Data Values file.

## Author contributions

AGDR carried out the majority of experiments, contributed to experimental design, and drafted the manuscript. EPF, LEM, AAV, BJL, PVB, SRZ, ABM, SYL, ADB, and AB executed some experiments and contributed ideas. EPF, AMA, and AGDR performed computational and statistical analyses. AP contributed to experimental design, wrote the manuscript, and administered the project. All authors reviewed the manuscript.

## Supplementary Material

Supplemental data

Supplemental tables 1-8

Supporting data values

## Version 1 09/14/2023

In-Press Preview

## Version 2 10/23/2023

Electronic publication
