# Supplementary material for: Factor-inhibiting HIF (FIH) promotes lung cancer progression
Source: JCI Insight. 2023 Oct 23;8(20):e167394. doi: 10.1172/jci.insight.167394 (PMC10619494; doi:10.1172/jci.insight.167394)

Supplemental figures

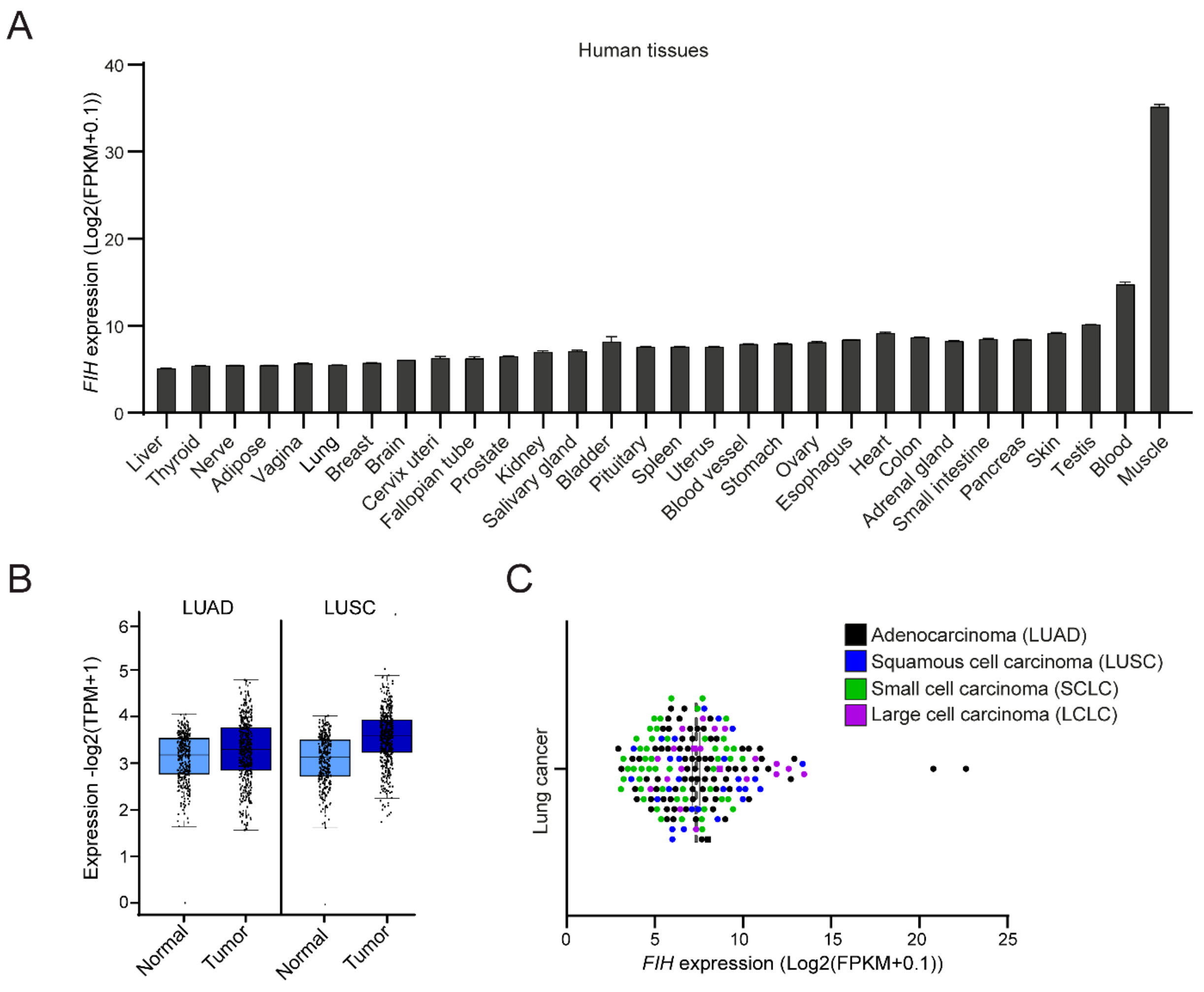

Supplemental Figure 1. *FIH* is expressed in both normal and malignant lung tissues. (A) *FIH* expression across human tissues corresponding to the indicated organs. Data from GTEx database was represented. **(B)** *FIH* is expressed in solid tumors: lung adenocarcinoma (LUAD, left) and lung squamous cell carcinoma (LUSC, right). Data was generated using GEPIA. **(C)** *FIH* expression across lung cancer cell lines corresponding to adenocarcinoma (LUAD, black), squamous cell carcinoma (LUSC, blue), small cell carcinoma (SCLC, green) or large cell carcinoma (LCLC, purple). Data was obtained from Omicsoft Studio, Qiagen.

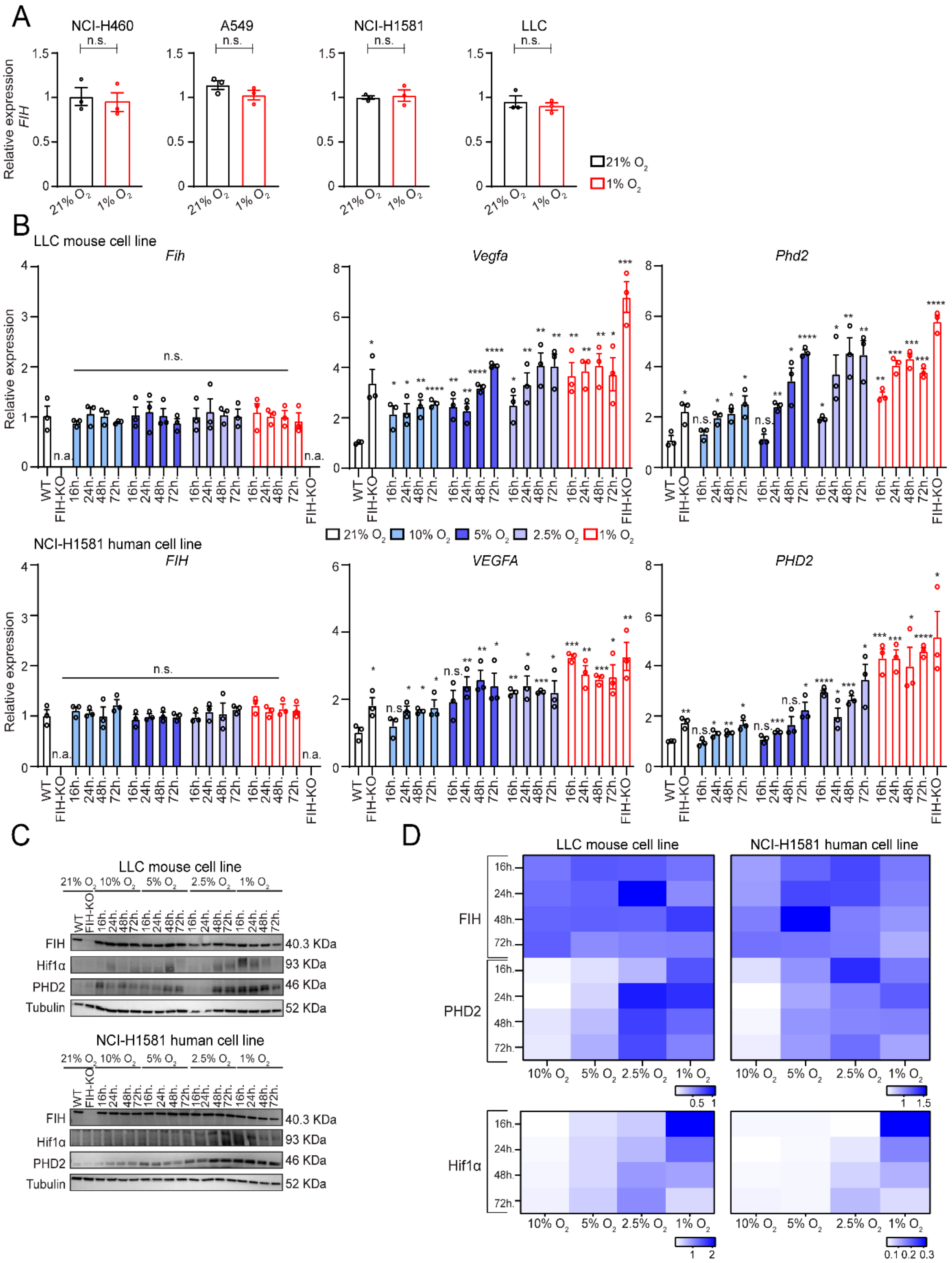

11

12 **Supplemental Figure 2. Hypoxia does not influence *FIH* expression in lung cancer cell**

13 **lines. (A)** Relative RNA expression levels of *FIH* after 16 hours of culture under normoxia

14 (black) or hypoxia (red) in lung cancer cell lines (n=3, unpaired t test). Data are presented as

15 mean ± SEM. n.s. (not significant). **(B)** Relative RNA expression levels of *FIH*, *VEGFA* and

PHD2 in LLC (top) or NCI-H1581 (bottom) cells cultured under normoxia (white) or the indicated levels of oxygen: 10% O_2 (blue), 5% O_2 (dark blue), 2.5% O_2 (purple) or 1% O_2 (red) at different time points: 16, 24, 48 or 72 hours. Data are presented as mean ± SEM (n=3, unpaired t test). Asterisks represent p values: * ($p \leq 0.05$), ** ($p \leq 0.01$), *** ($p \leq 0.001$) and **** ($p \leq 0.0001$). n.s. (not significant). n.a. (not amplification observed). **(C)** Western blot showing the levels of FIH, HIF1A, PHD2 or tubulin protein levels of WT or *FIH*-KO cells corresponding to LLC (top) or NCI-H1581 (bottom) cells cultured at the indicated time points. **(D)** Heatmap showing a direct comparison of the levels of FIH and PHD2 protein levels (top) and the levels of HIF1A (bottom). Quantifications were made by densitometric analysis of the immunoblots shown in C.

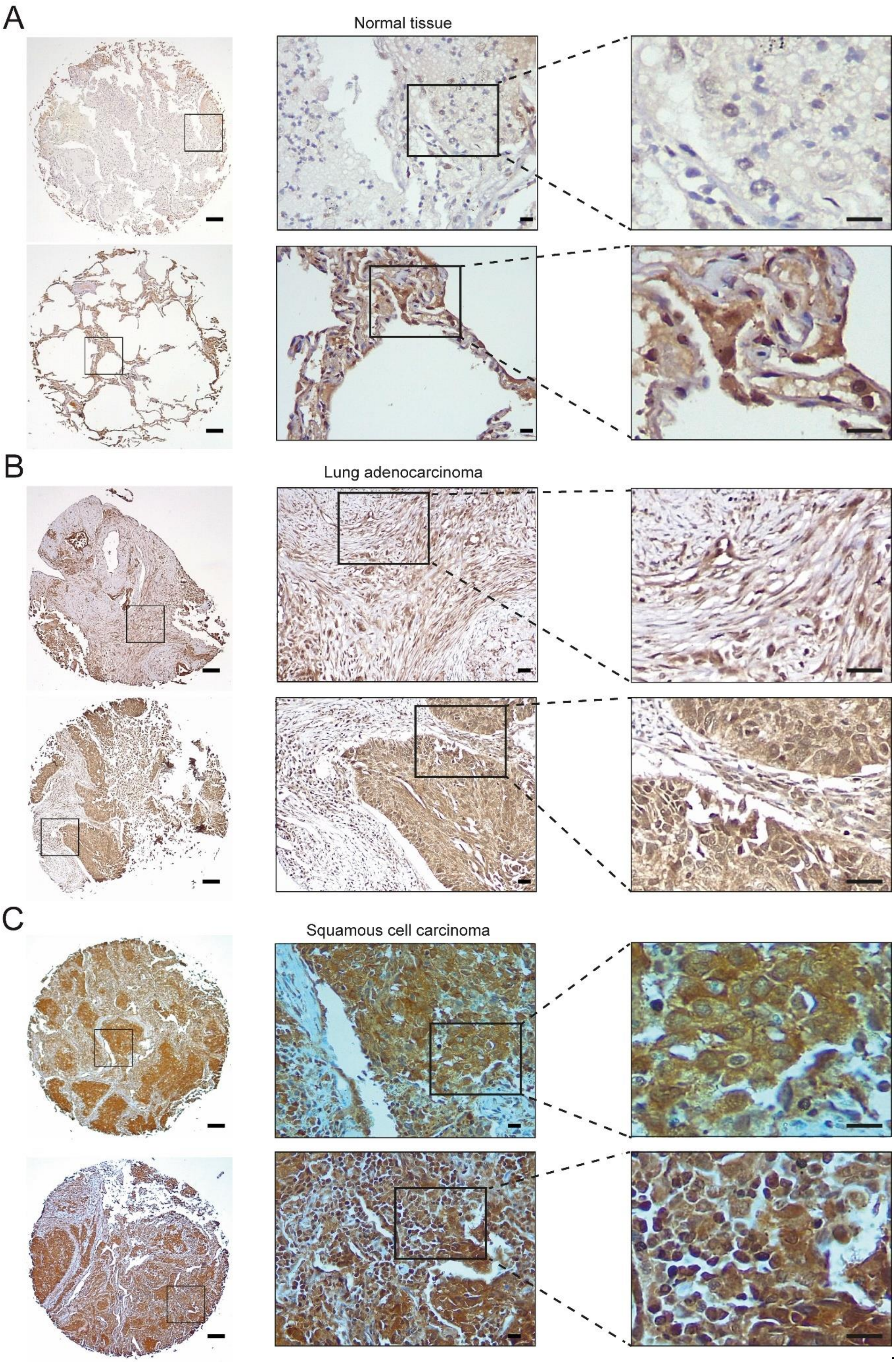
A
Normal tissue
B
Lung adenocarcinoma
C
Squamous cell carcinoma

Supplemental Figure 3. Expression of FIH in lung tissues. Representative immunohistochemistry images showing varying degrees of FIH (peroxidase-DAB brown staining) in lung cancer tissue microarrays in: **(A)** normal tissue, **(B)** lung adenocarcinoma (LUAD) or **(C)** squamous cell carcinoma (LUSC). Insets show image magnifications. Scale bars = 500 µm.

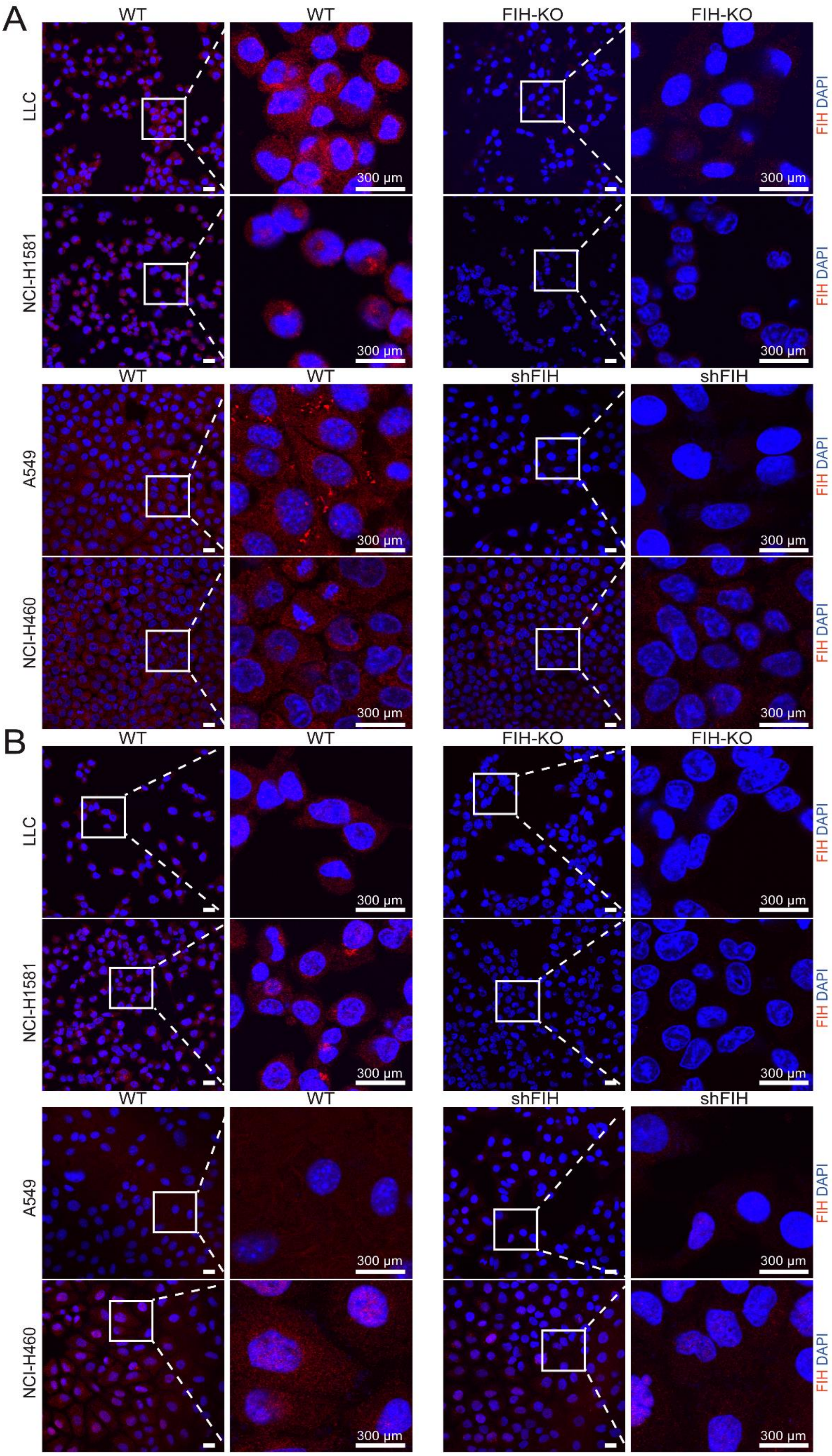
A
WT
WT
FIH-KO
FIH-KO
LLC
NCI-H1581
WT
WT
shFIH
shFIH
A549
NCI-H460
FIH DAPI
300 μm
B
WT
WT
FIH-KO
FIH-KO
LLC
NCI-H1581
WT
WT
shFIH
shFIH
A549
NCI-H460
FIH DAPI
300 μm

34

Supplemental Figure 4. FIH is mainly located in the cytoplasm compartment of lung cancer cells. Immunofluorescence showing FIH expression levels (red) and DAPI (blue) in WT or *FIH*-KO for the indicated cell lines (LLC, NCI-H1581, A549 and NCI-H460) after 16 hours of culture in: **(A)** normoxia or **(B)** hypoxia. Insets show image magnifications for each condition. Scale bars = 300 µm.

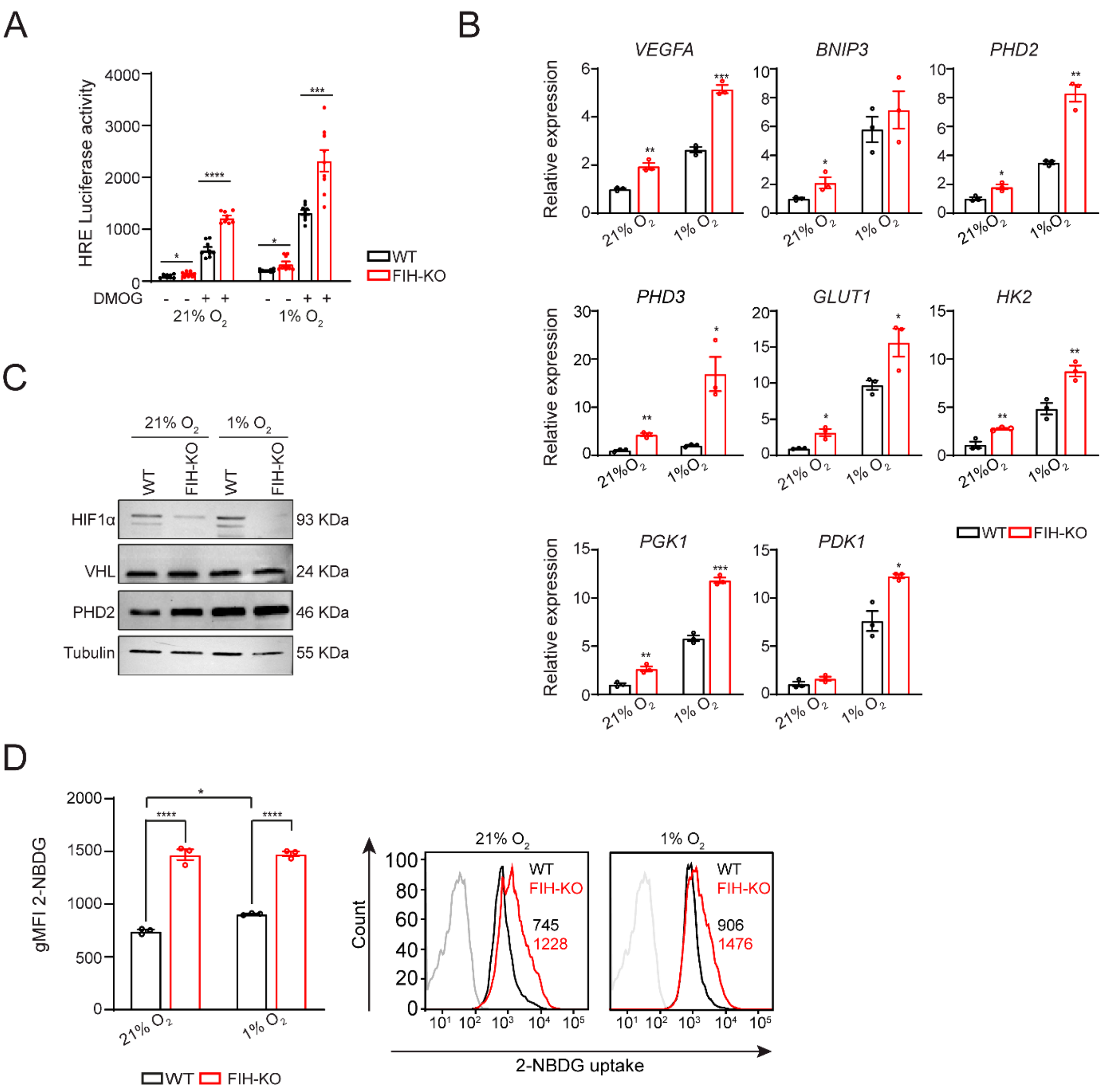

Supplemental Figure 5. *FIH* deletion in lung cancer cells promotes HIF-driven metabolism. **(A)** HRE luciferase reporter activity assay performed with NCI-H1581 cells cultured in the presence or absence of 1mM DMOG under the indicated oxygen levels. (n=3, technical replicates are shown, unpaired t test). **(B)** Relative expression of the indicated HIF-target genes in WT (black) or *FIH*-KO (red) NCI-H1581 cells after 16 hours of culture under normoxia or hypoxia, measured by qPCR (n=3, unpaired t test). **(C)** Immunoblotting showing HIF1A, PHD2, VHL and tubulin expression in WT or *FIH*-KO NCI-H1581 cells, cultured under normoxia or hypoxia for 16 hours. **(D)** Flow cytometry analysis of glucose uptake (2-NBDG) in WT (black) or *FIH*-KO (red) NCI-H1581 cells cultured under normoxia or hypoxia for 48 hours. A representative histogram with gMFI values for each condition (unstained, grey; WT, black or *FIH*-KO, red) is shown (n=3, two-way ANOVA). Data are presented as mean ± SEM. Asterisks represent p values: * ($p \leq 0.05$), ** ($p \leq 0.01$), *** ($p \leq 0.001$) and **** ($p \leq 0.0001$).

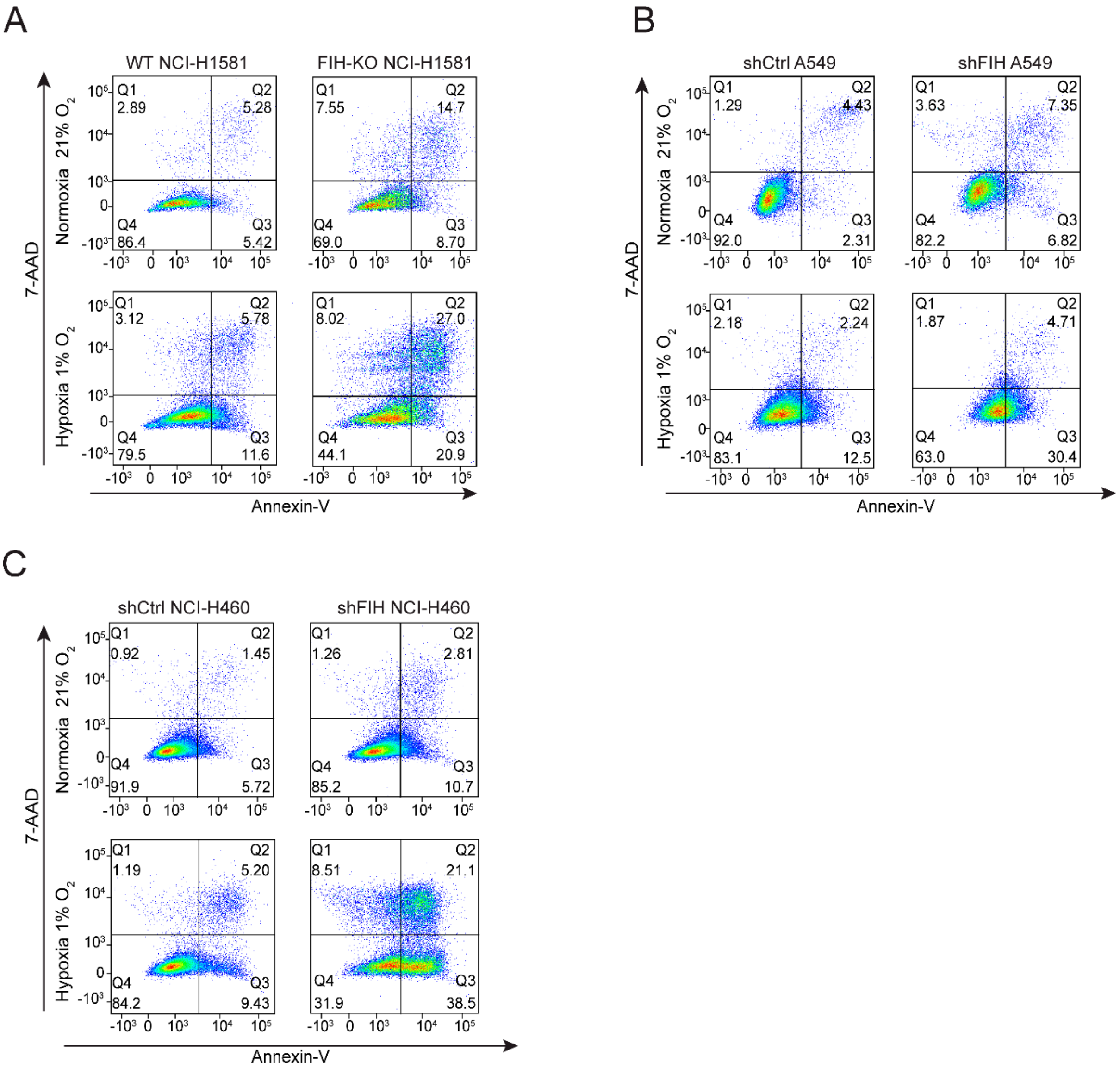

Supplemental Figure 6. ***FIH* deletion in tumor cells impairs cell survival in vitro. (A-C)** Representative flow cytometry dot plots corresponding to human lung cancer cell line NCI-H1581 (A), A549 (B) and NCI-H460 (C) stained with 7-AAD and Annexin-V. The percentage values of early (Q3) or late (Q4) apoptotic cells are shown in each panel.

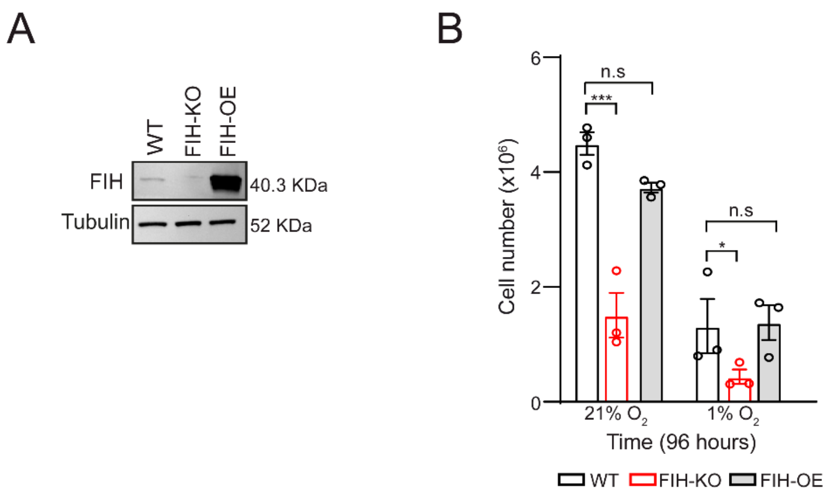

Supplemental Figure 7. Overexpression of *FIH* in LLC cells prevents HIF activity and does not impact cell proliferation. (A) Western blot showing the levels of FIH and tubulin in WT, *FIH*-KO or *FIH*-overexpressing (*FIH*-OE) LLC cells. **(B)** Number of cells of WT (black), *FIH*-KO (red) or *FIH*-OE (grey) LLC cells cultured under normoxia or hypoxia as indicated. (n=3, one-way ANOVA). Data are presented as mean ± SEM. Asterisks represent p values: * ($p \leq 0.05$) and *** ($p \leq 0.001$). n.s. (not significant).

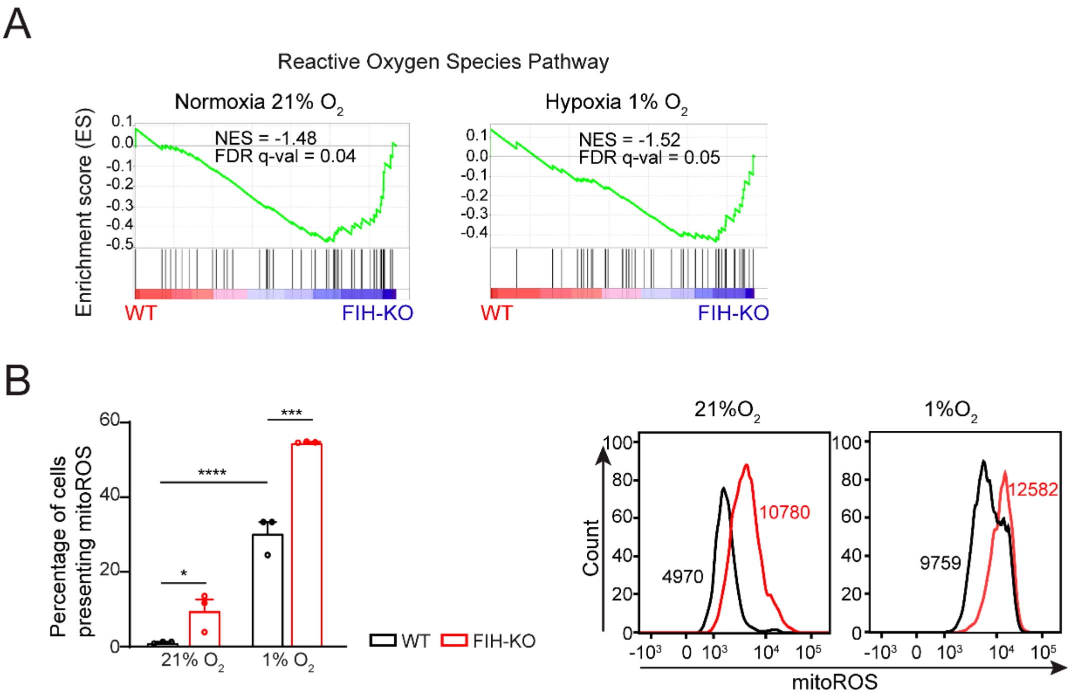

Supplemental Figure 8. *FIH* loss promotes a significant increase in ROS levels. (A) Individual gene set enrichment GSEA plots corresponding to the reactive oxygen species pathway in WT LLC cells in comparison to *FIH*-KO LLC cells, cultured in normoxia (right) or hypoxia (left). Normalized Enrichment score (NES) and FDR q values are shown. **(B)** Bar graphs representing percentage of cells (left) and gMFIs (right) of WT (black) or *FIH*-KO (red) LLC cells stained with mitoSOX; cells were cultured under normoxia or hypoxia for 48 hours. A representative flow cytometry plot for each condition is shown (n=3, two-way ANOVA). Data are presented as mean ± SEM. Asterisks represent p values: * ($p \leq 0.05$) and *** ($p \leq 0.001$).

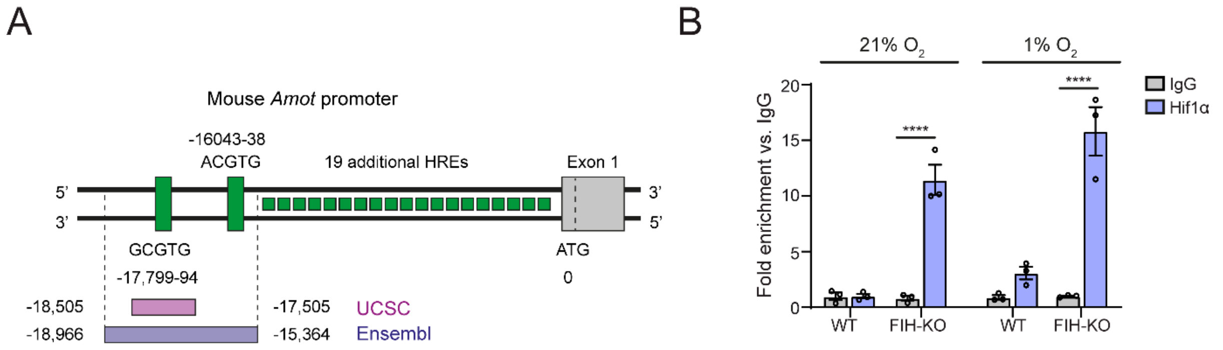

Supplemental Figure 9. Characterization of functional hypoxia-response elements (HREs) in the promoter region of the *Amot* gene. (A) Positions of putative identified HRE motifs in the promoter region of the *Amot* gene predicted by UCSC Genome Browser (pink) and Ensembl (purple). The number of additional HREs between the promoter and the starting codon (ATG) is also shown. **(B)** ChIP-qPCR values representing the fold enrichment of HIF1A binding to the indicated HRE motifs in the Amot promoter in mouse LLC cell lines cultured under normoxia or hypoxia for 16 hours. Control ChIP was performed with IgG (n=3, two-way ANOVA with Bonferroni correction). Data are represented as mean ± SEM. Asterisks represent p values: **** ($p \leq 0.0001$).

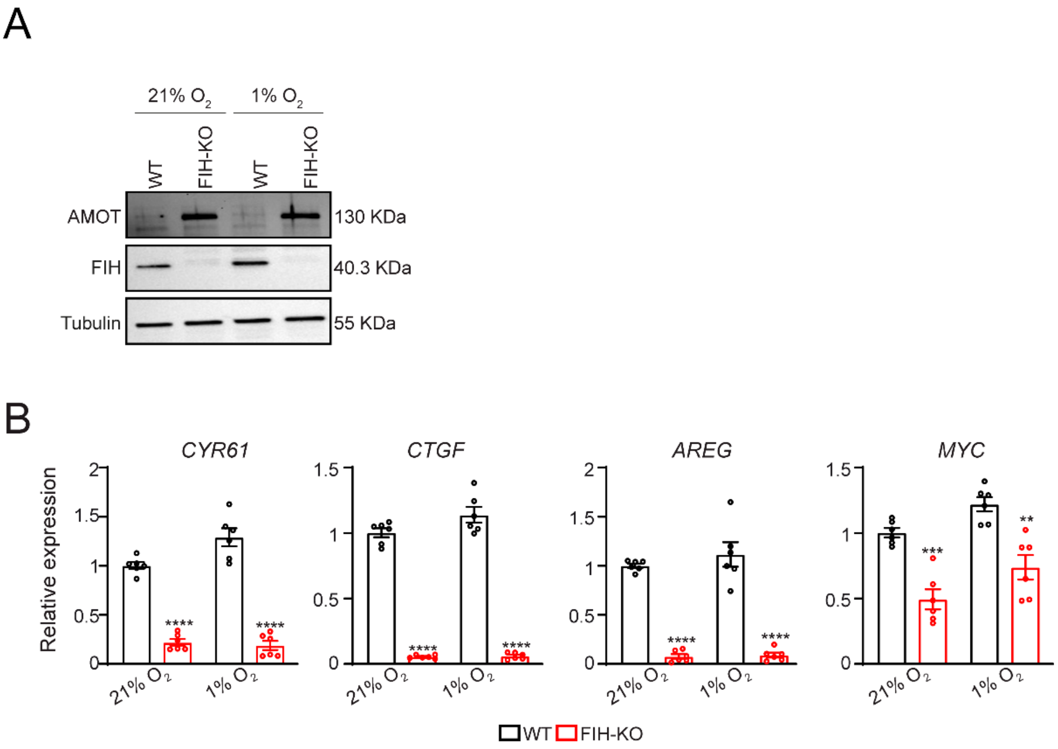

Supplemental Figure 10. FIH deletion results in an increase in Amot expression and inhibition of YAP/TAZ signaling in human lung cancer cells. (A) Western blot showing the levels of AMOT, FIH and tubulin in WT or *FIH*-KO NCI-H1581 cells after 16 hours of culture under normoxia or hypoxia. **(B)** Relative RNA expression of *CYR61*, *CTGF*, *AREG* and *MYC* genes in NCI-H1581 WT or *FIH*-KO cells cultured under normoxia or hypoxia, measured by qPCR (n=3, technical replicates are shown, unpaired t test). Data are presented as mean ± SEM. Asterisks represent p values: ** ($p \leq 0.01$), *** ($p \leq 0.001$) and **** ($p \leq 0.0001$).

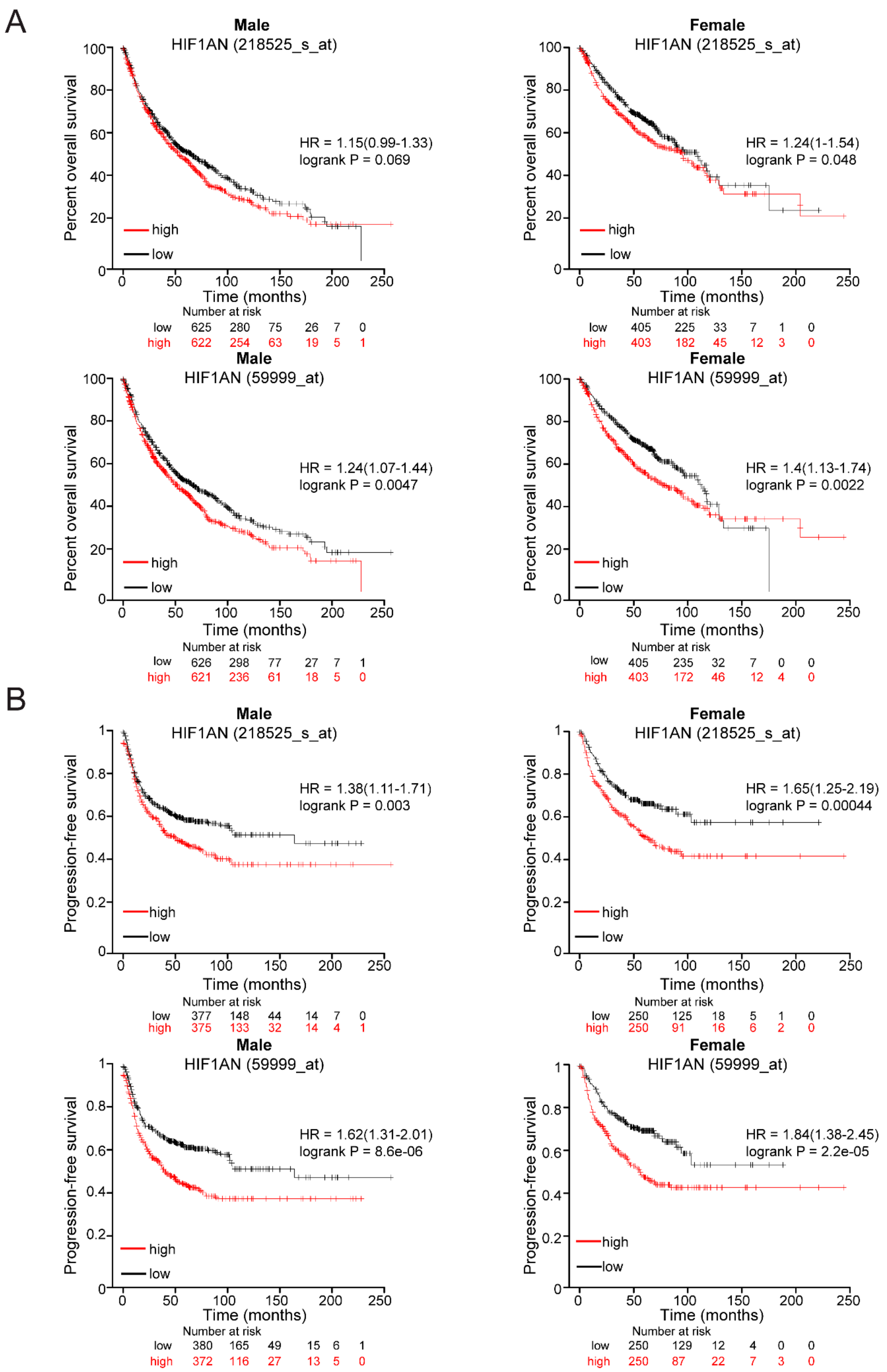
A
Male
HIF1AN (218525_s_at)
HR = 1.15(0.99-1.33)
logrank P = 0.069
Percent overall survival
Time (months)
high
low
Number at risk
low 625 280 75 26 7 0
high 622 254 63 19 5 1
Female
HIF1AN (218525_s_at)
HR = 1.24(1-1.54)
logrank P = 0.048
Number at risk
low 405 225 33 7 1 0
high 403 182 45 12 3 0
Male
HIF1AN (59999_at)
HR = 1.24(1.07-1.44)
logrank P = 0.0047
Number at risk
low 626 298 77 27 7 1
high 621 236 61 18 5 0
Female
HIF1AN (59999_at)
HR = 1.4(1.13-1.74)
logrank P = 0.0022
Number at risk
low 405 235 32 7 0 0
high 403 172 46 12 4 0
B
Male
HIF1AN (218525_s_at)
HR = 1.38(1.11-1.71)
logrank P = 0.003
Progression-free survival
Number at risk
low 377 148 44 14 7 0
high 375 133 32 14 4 1
Female
HIF1AN (218525_s_at)
HR = 1.65(1.25-2.19)
logrank P = 0.00044
Number at risk
low 250 125 18 5 1 0
high 250 91 16 6 2 0
Male
HIF1AN (59999_at)
HR = 1.62(1.31-2.01)
logrank P = 8.6e-06
Number at risk
low 380 165 49 15 6 1
high 372 116 27 13 5 0
Female
HIF1AN (59999_at)
HR = 1.84(1.38-2.45)
logrank P = 2.2e-05
Number at risk
low 250 129 12 4 0 0
high 250 87 22 7 3 0

Supplemental Figure 11. High FIH expression correlates with poor overall and progression-free survival in male and female NSCLC patients. (A) Kaplan-Meier overall survival plots of lung cancer patients (n=2055) classified by gender: male (n=1247, left panels) and female (n=808, right panels). A log rank test for each panel was performed and p values are indicated. **(B)** Progression-free survival plots of lung cancer patients (n=1252) classified by gender: male (n=500, left panels) and female (n=752, right panels). Affymetrix gene IDs are indicated in each plot.

Full unedited gel for Figure 1B

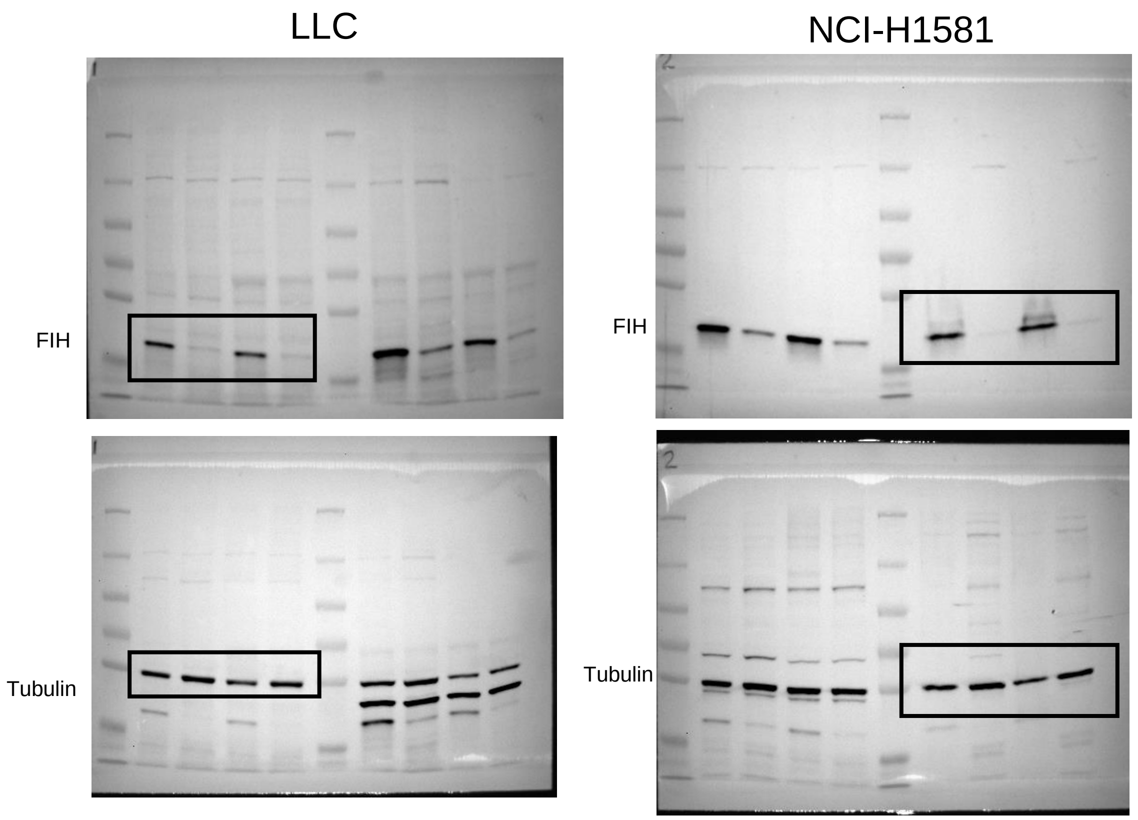

Full unedited gel for Figure 1C

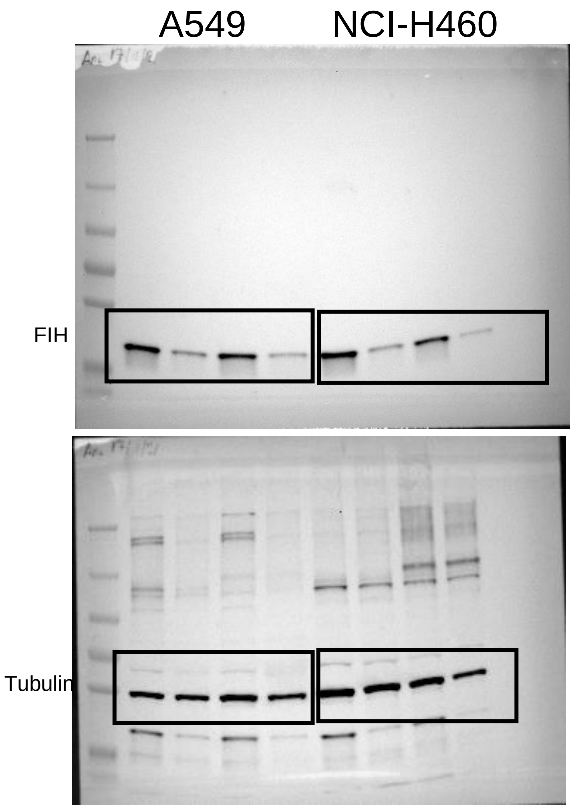

Full unedited gel for Figure 1F

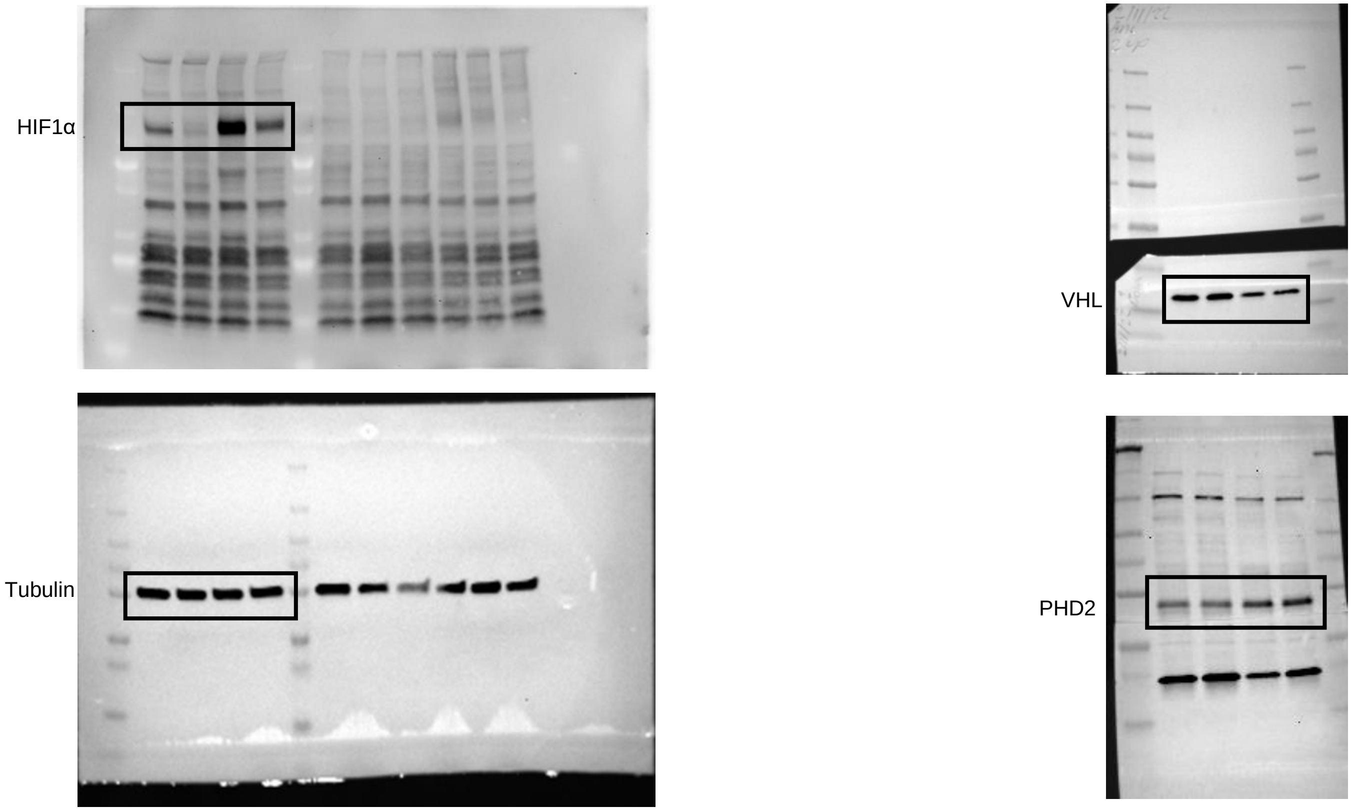

Full unedited gel for Figure 2C

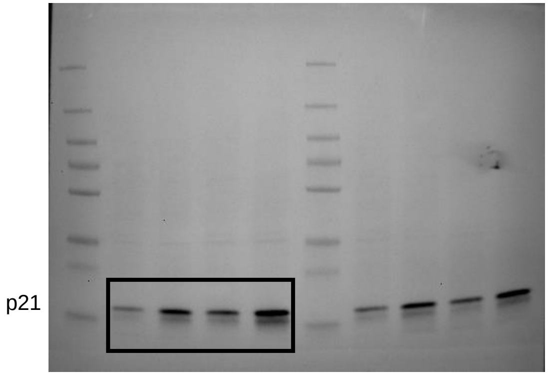

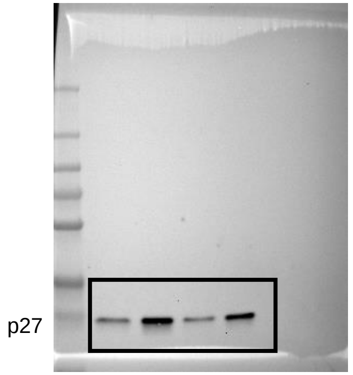

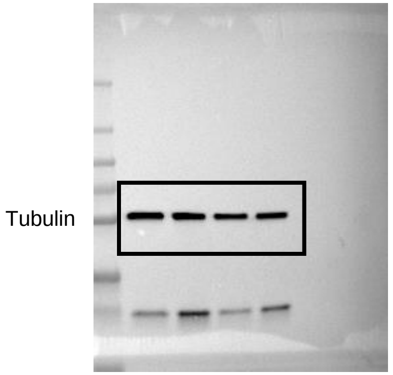

Full unedited gel for Figure 4B

Amot

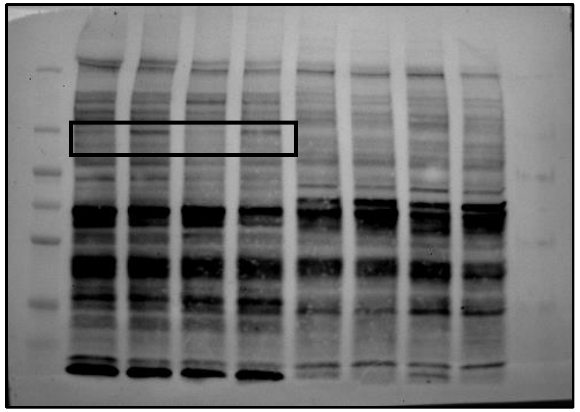

Tubulin

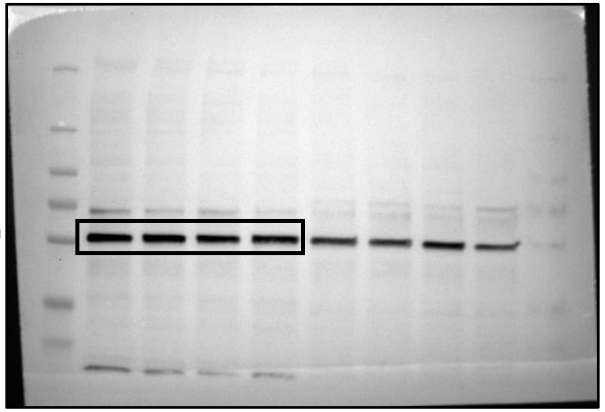

Full unedited gel for Figure 4F

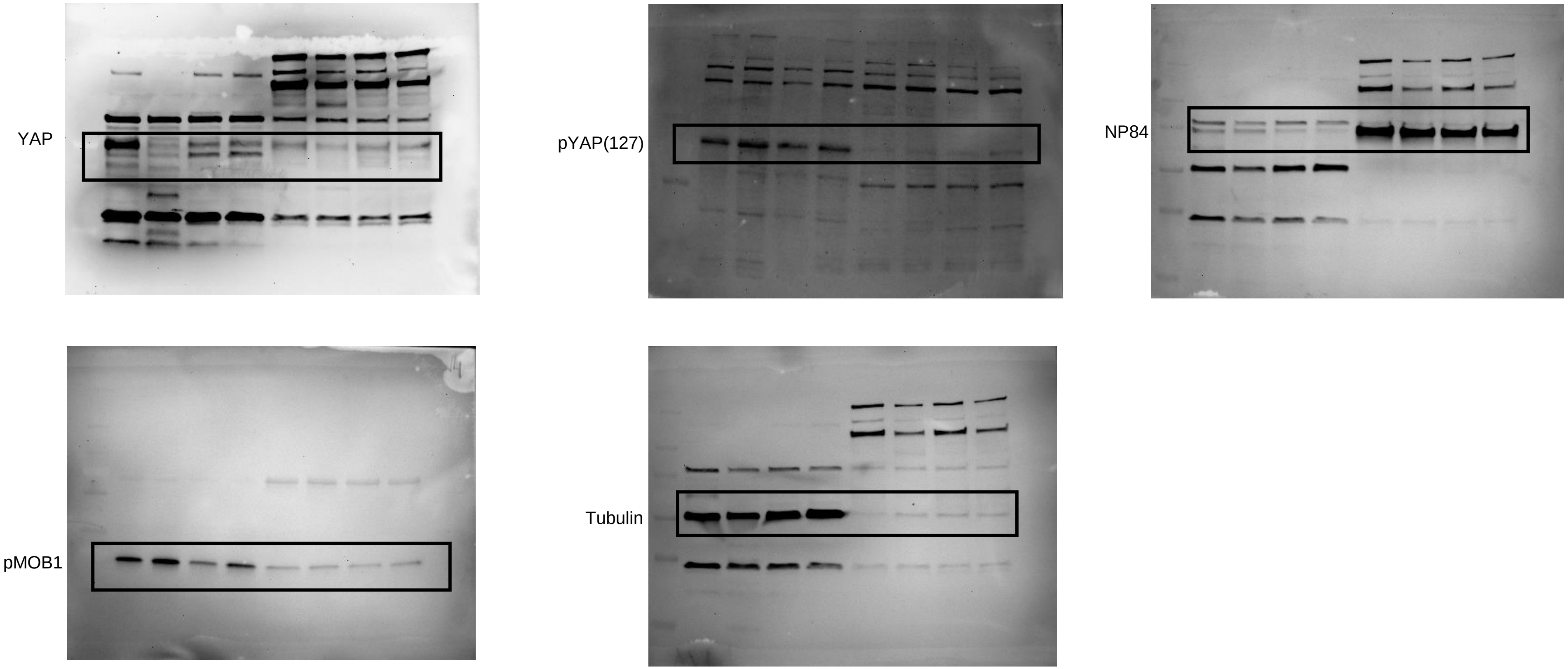

Full unedited gel for Figure 5A

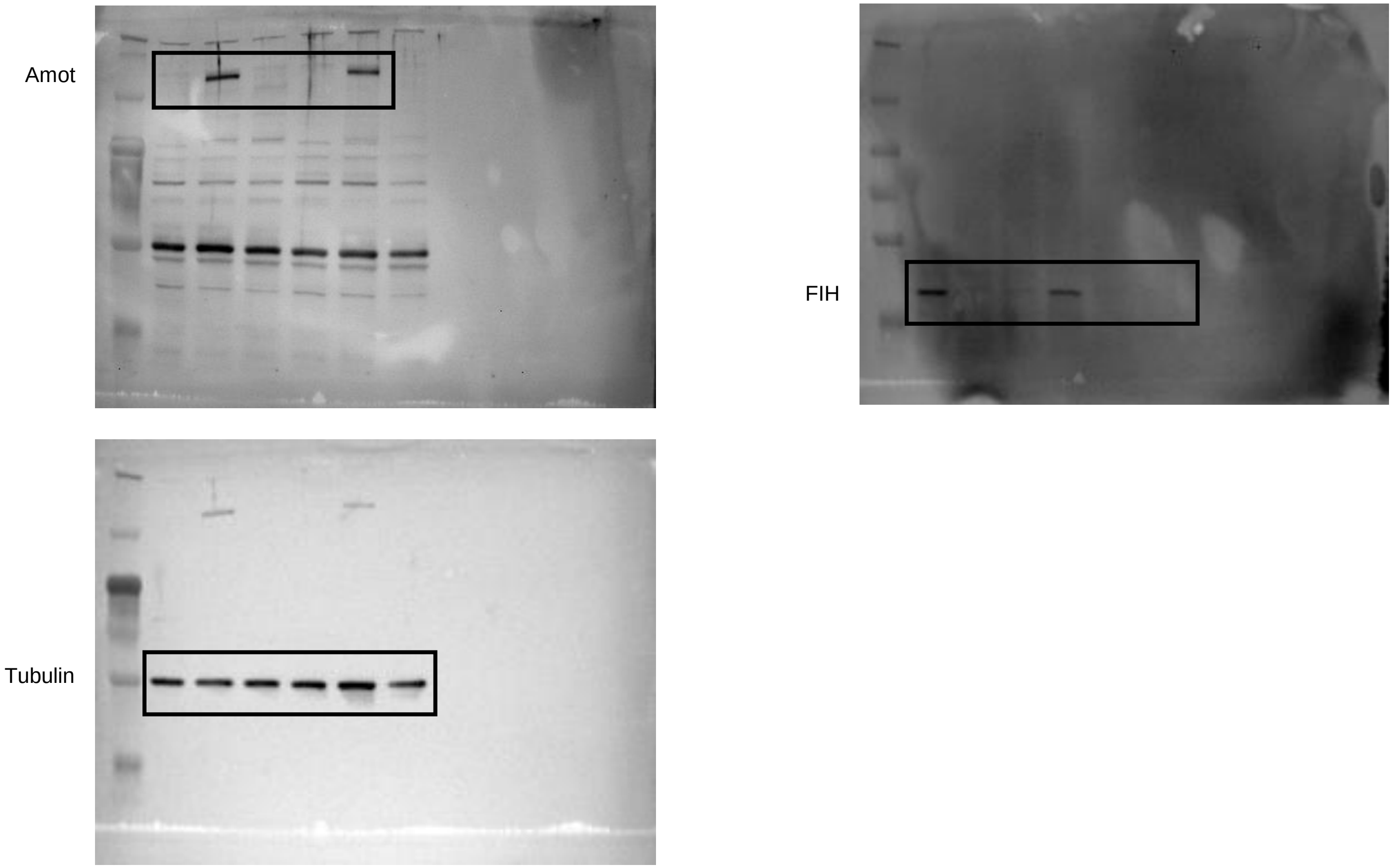

Full unedited gel for Supplemental Figure 2C

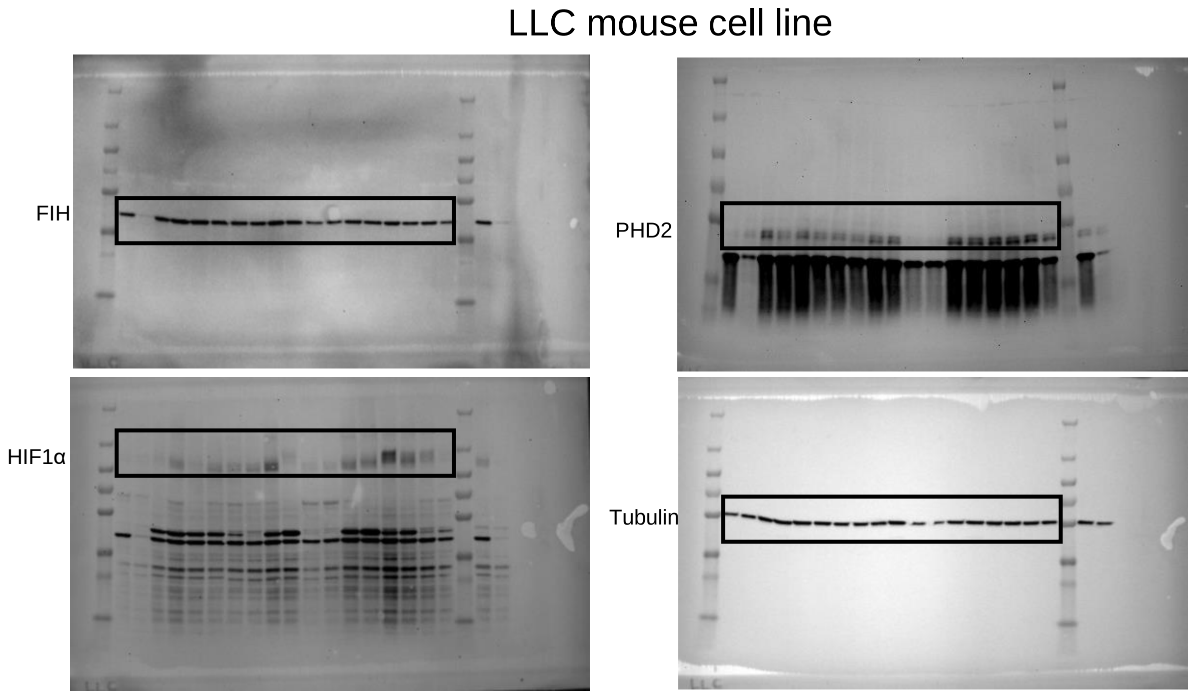

Full unedited gel for Supplemental Figure 2C

NCI-H1581 human cell line

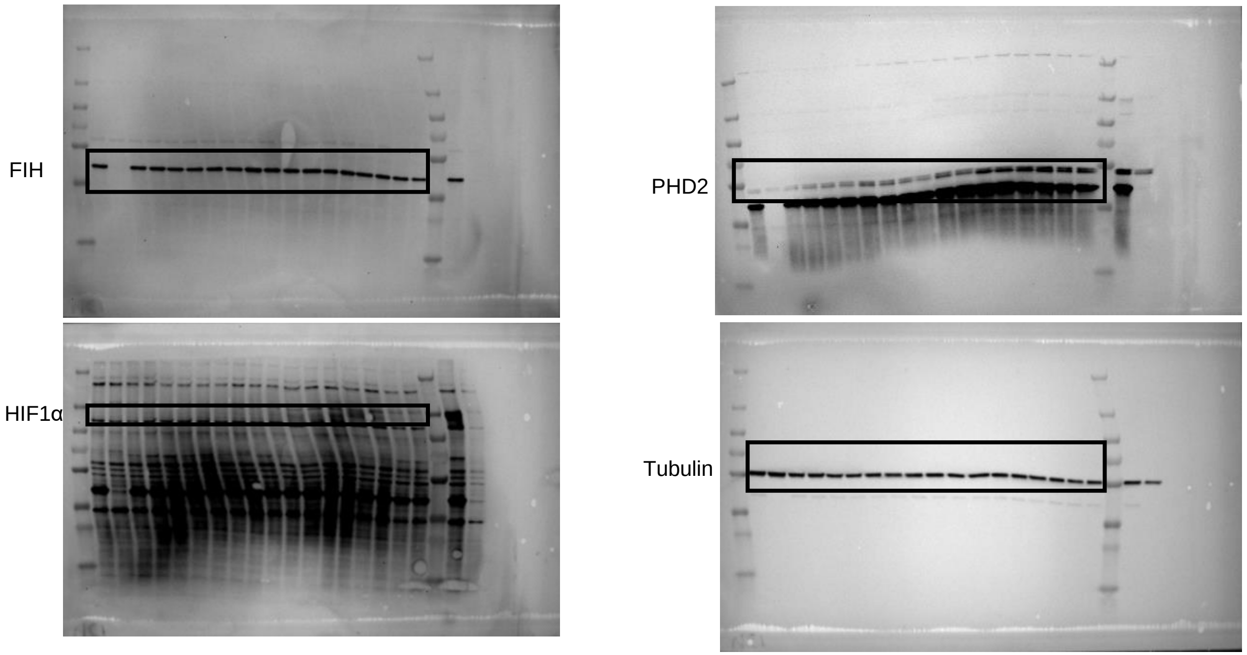

Full unedited gel for Supplemental Figure 5C

HIF1a

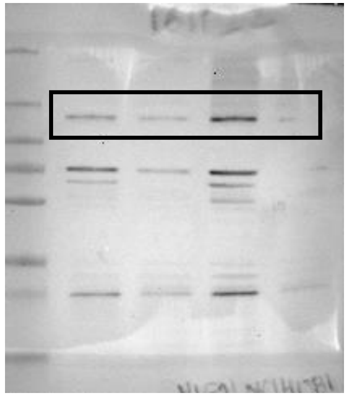

PHD2

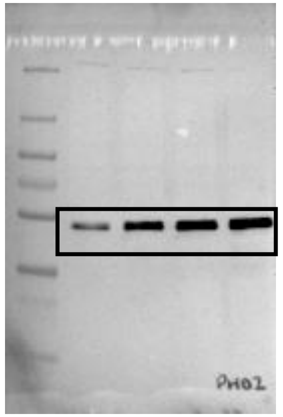

VHL

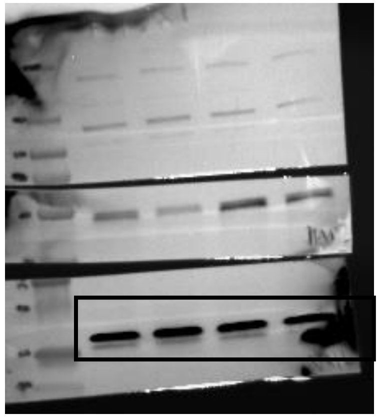

Tubulin

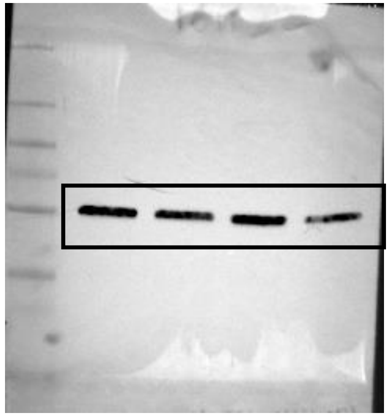

Full unedited gel for Supplemental Figure 7A

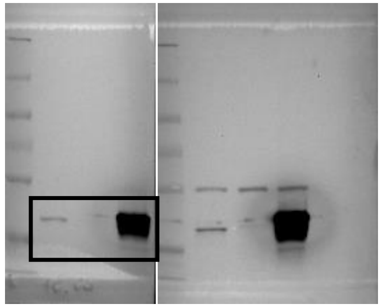

FIH

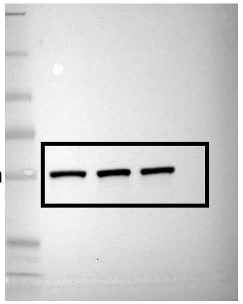

Tubulin

Full unedited gel for Supplemental Figure 10A

Amot

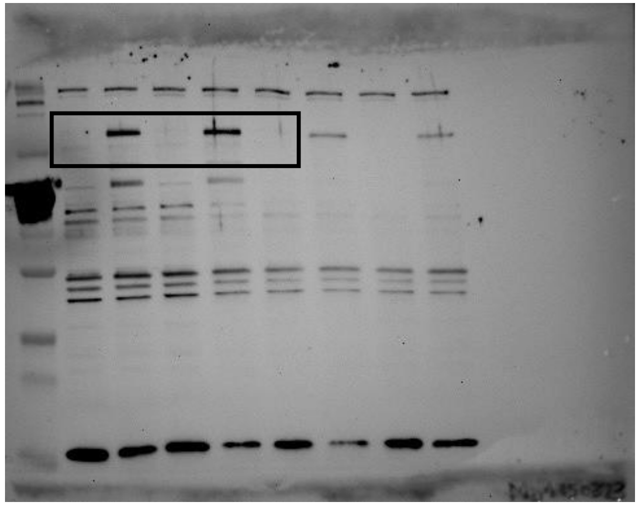

Tubulin

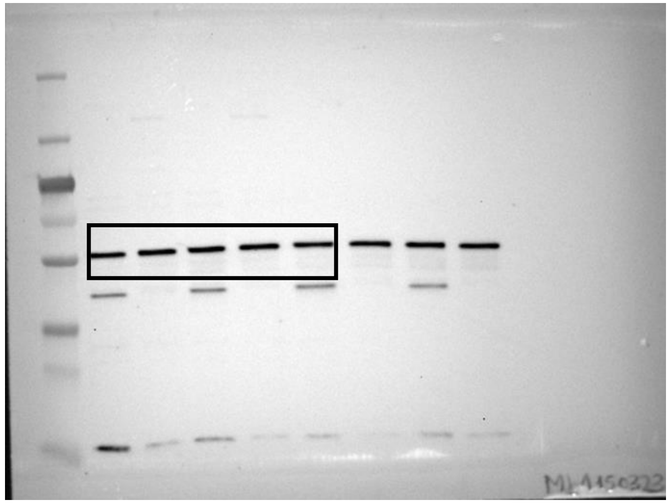

FIH

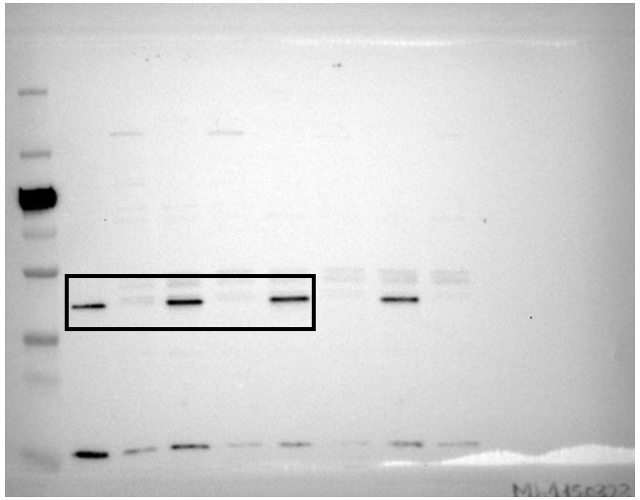

Supplement: Supplemental data [file jciinsight-8-167394-s139.pdf]
